# Hierarchical Manufacturing of Anisotropic and High-Efficiency Electromagnetic Interference Shielding Modules for Smart Electronics

**DOI:** 10.1007/s40820-025-02024-4

**Published:** 2026-01-05

**Authors:** Shaohong Shi, Siwen Deng, Yuheng Jiang, Jiabin Chen, Lukas Sporrer, Fangchao Cheng, Quanquan Guo, Jingjing Jing, Yinghong Chen

**Affiliations:** 1 Guangxi Key Laboratory of Processing for Non-ferrous Metals and Featured Materials, School of Resources, Environment and Materials, Guangxi University, No. 100, Daxuedong Road, Nanning, 530004 People’s Republic of China; 2 Max Planck Institute of Microstructure Physics, Weinberg 2, 06120 Halle (Saale), Germany; 3 State Key Laboratory of Polymer Materials Engineering, Polymer Research Institute of Sichuan University, Sichuan University, No. 24 South Section 1, Yihuan Road, Chengdu, 610065 People’s Republic of China

**Keywords:** 3D printing, Graphene nanoparticles, Hierarchical manufacturing, Electromagnetic interference shielding, Thermal management

## Abstract

To shield electronics from complicated electromagnetic environments caused by wireless electromagnetic waves, achieving elaborately structural manufacturing while not sacrificing electromagnetic interference shielding performances remains crucial challenges. Herein, we propose a hierarchical manufacturing method that combines the use of 3D printing shear flow field and layer-by-layer assembly for fabricating the structurally customizable and multifunctional polylactic acid@graphene nanoparticle (PLA@GNs) materials. The dynamic behavior of polymer fluids is firstly explored via computational fluid dynamic simulation, and a Weissenberg number is employed to quantitatively analyze the disordered-to-ordered structural evolution of molecular chains and nanoparticles, allowing to tailor the micro-scale ordered structures. Subsequently, the macro-scale 3D architectures of PLA@GNs modules are fabricated by layer-by-layer assembly. Owing to the aligned GNs, the shielding performance reaches 41.2 dB, simultaneously accompanied by a directional thermal conductivity of 3.2 W m^−1^ K^−1^. Moreover, the potential application of 3D-printed shielding modules in specific civilian frequency bands such as 4G (1800–2100 MHz), Bluetooth (2402–2480 MHz), and 5G (3300–3800 MHz) is fully demonstrated. Overall, this work not only establishes a universal methodology about 3D printing shear flow field-driven orientation of two-dimensional nanoparticles within polymer fluids, but also gives a scientific method for advanced manufacturing of the next-generation electromagnetic functional modules for smart electronics.

**Supplementary Information:**

The online version contains supplementary material available at 10.1007/s40820-025-02024-4.

## Highlights

- A universal methodology regarding 3D printing shear flow field-driven orientation of two-dimensional graphene nanoparticles within polymer fluids is established *via* computational fluid dynamics simulation.

- A hierarchical manufacturing strategy is purposed to assembly polylactic acid@graphene nanoparticle (PLA@GNs) modules with anisotropic structural characteristics for contributing on the electromagnetic compatibility and heat dissipation of electronics.

- The exceptional functionalities of 3D-printed modules including high-efficiency Electromagnetic shielding performance (41.2 dB) and directional thermal conductivity (3.2 W m^−1^ K^−1^) are achieved.

**Supplementary Information:**

The online version contains supplementary material available at 10.1007/s40820-025-02024-4.

## Graphical abstract

**Figure:**
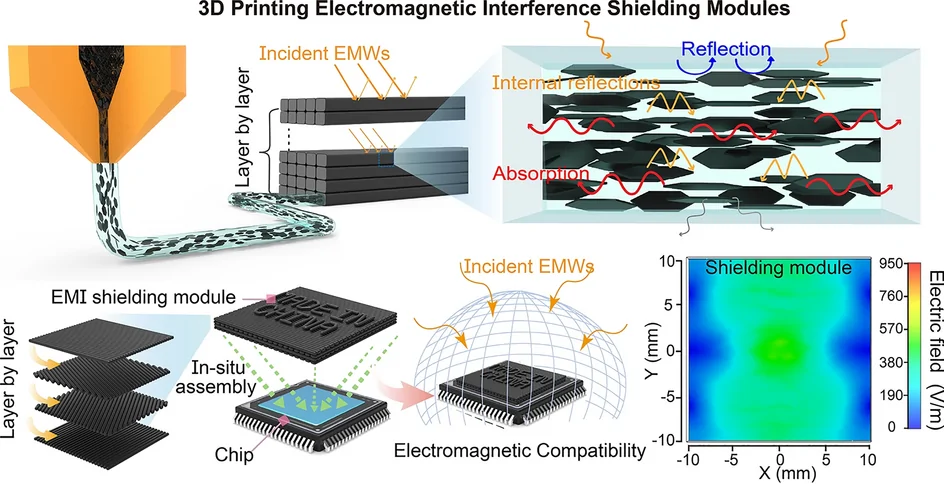

## Introduction

With the continuous development of 5G/5G-advanced wireless networks, the increasing electromagnetic radiation derived from high-frequency/high-power wireless electromagnetic waves (EMWs) has become a crucial issue for electronic safety and human health [1–3]. Electromagnetic compatibility (EMC) is a scientific approach to avoid electromagnetic interference and realize normal operation of electronic systems in electromagnetic environments [4–6]. At present, commercial metal-based shielding modules are commonly employed to meet EMC requirements of electronics, widely applied in fields such as artificial intelligence, human–machine interactions, and telemedicine systems, etc. [7–9]. However, traditional metal-based modules are facing difficulties, toward further miniaturization intelligent and integrated electronics, severely restricting their further development: (1) extremely complex manufacturing procedures based on traditional processing techniques such as electroplating, spraying, and sputtering, leading to long production times and high costs; (2) obstacles to large-scale manufacturing of shielding units for electronics with meticulous, custom-tailored, and conformal characteristics; (3) inherent drawbacks of metals including their high density, processing difficulties and susceptibility to environments [10]. Hence, there is an urgent need to introduce novel materials and advanced technologies to develop new-generation shielding modules with customized architectures and high-efficiency shielding performance, to allow their application in miniaturized, integrated, and intelligent electronics.

To solve the existing problems, a variety of new materials, including MXenes, ferromagnets, and carbon nanomaterials, have been investigated to potentially replace metal-based modules [11–13]. Among these material categories, graphene and its derived composites offer a multitude of possibilities for the fabrication of advanced shielding materials owing to their light weight, multifunctionality, commercial availability, and scalable production [14–16]. Some reported literature on graphene-based shielding materials have fully demonstrated their positive contribution to the EMC demands of electronics [17, 18]. Due to the abundant conduction electrons of graphene nanoparticles, the electromagnetic energy is efficiently dissipated through the directional migration of electrons along the local micro-current network and the transfer between graphene lamellae [18, 19]. Recently, assembling the anisotropic ordered structures of graphene lamellae driven by external fields (electro, magnetic, and electromagnetic fields) has well demonstrated the feasibility for realizing the expecting functionalities in specific dimensionalities, e.g., shielding, thermal conductivity, etc. [20, 21]. Nevertheless, the as-mentioned field-driven orientation strategies in practical applications are still constrained by the complexities involved in the fabrication, the limited scalability, and the challenge for customizing the geometric shapes of modules. Detailedly, (1) the high cost for manipulating micro-scale ordered structures by electro/magnetic fields, (2) the complicated procedures for designing macroscopic shielding units with anisotropic microstructures, and (3) the restricted space in electronics leading to the theory–practice gap in assembling the shielding units with large-scale, customizable, and conformal characteristics.

Compared with the fabrication of shielding materials by state-of-the-art methods, fused deposition modeling (FDM) 3D printing, as an additive manufacturing technology, possesses the overwhelming superiority to fabricate customizable geometries with complicated 3D architectures [22, 23]. Herein, thanks to the rich experience in 3D printable polymer-based composites [24–26], a hierarchical manufacturing strategy is demonstrated by tailoring micro-scale graphene ordered structures and macro-scale 3D architectures, to obtain anisotropic and high-efficiency shielding modules for electronics. Notably, the environmentally friendly, sustainable, and shear-dependent polymer of bio-based polylactic acid (PLA), as well as the typical commercial two-dimensional nanomaterials of graphene nanoparticles (GNs), is selected as the ideal printable matrix and fillers, respectively, to investigate the orientation dynamics in the polymer/two-dimensional nanomaterial systems. The successful implementation is beneficial to building a universal methodology for tailoring the microstructure of multidimensional nanoparticles within polymer matrix via shear flow field, and achieving the expecting functions for materials, thereby enabling potential applications. Initially, the effect of the shear flow field on the microstructural evolution of the PLA chains and the coupled GNs in 3D printing liquefier channels is systematically explored through computational fluid dynamic (CFD) simulation, thereby establishing the formation mechanism of micro-scale ordered GNs structures. Following the determined relationships, the macro-scale 3D architectures are progressively assembled in a layer-by-layer stacking manner, thus achieving the hierarchical manufacturing of anisotropic and functional modules for electronics. The electromagnetic interference (EMI) shielding performance of 3D-printed PLA@GNs samples reaches 41.2 dB, simultaneously accompanied by a directional thermal conductivity of 3.2 W m^−1^ K^−1^, which is mainly attributed to the well-tailored ordered GNs structures. Overall, the universal methodology established in this work regarding the shear flow field-driven orientation of two-dimensional nanoparticles within polymer matrix would greatly expand the application potentials of this approach to other engineering plastics (e.g., polyamide, polyetheretherketone) and two-dimensional nanomaterials (e.g., MXene, boron nitrides), thereby laying the foundation for manufacturing the next-generation functional electronics.

## Experimental Section

### Materials

Polylactic acid particles (PLA, Ingeo 6201D), with a specific gravity 1.24 g cm^−3^ and a melt flow rate 15–30 g/10 min, were supplied by NatureWorks LLC, USA. Graphene nanoparticles (GNs, XFQ022) as multifunctional additives were purchased by Nanjing XFNANO Material Technology Co. Deionized water was supported by Kelong Chemical Reagent Factory, China. All chemical materials were used without purification.

### Fabrication of PLA@GNs Filaments and 3D-Printed Models

A certain amount of GNs (2.5, 5, 7.5, and 10 g) were dispersed in deionized water by vigorous stirring combined with ultrasonication for 30 min to achieve uniform dispersion. After a stable suspension was obtained, 50 g PLA was gradually and slowly added, and the dispersed process was continued for 30 min, which laid a good foundation for homogeneous coating of GNs nanoparticles onto the surface of PLA spheres. The mixture was then filtered using a Brinell funnel. Afterward, it was dried at 80 °C for 8 h. The PLA@GNs filaments suitable for 3D printing were extruded at a temperature of 180 °C and a screw speed of 10 rpm using a screw extruder (model: RM-200C) manufactured by Harbin Hai Pu Electric Technology Co. The resulting composites were labeled with PLA@GNs filaments, and the detailed component is also provided in Table S1.

The obtained filaments were printed by fused deposition modeling (FDM) printer (RepRap X400, Feldkirchen, Germany) on slices of the digital model according to the Simplify 3D software to produce samples with different characterizations. The optimized printing parameters are comprised: 0.4 mm nozzle diameter, 190 °C printing temperature, 0.30 mm layer height, and 100% rectilinear infill density. Owing to the effect of printing speed onto the flow behaviors of polymer fluids, the FDM printing speeds were selected to be 600, 2400, 3600, and 4800 mm min^−1^, which is corresponding to the different volume flow rates of 1.15, 4.60, 6.88, and 9.20 mm^3^ s^−1^. Finally, the printing models were printed and assembled as the preset trajectories. The test samples used to characterize EMI shielding and thermal conductivity were prepared at a volume flow rate of 9.20 mm^3^ s^−1^.

## Results and Discussion

### Disordered-to-Ordered Structural Evolution of Molecular Chains and Nanoparticles

**Fig. 1:**
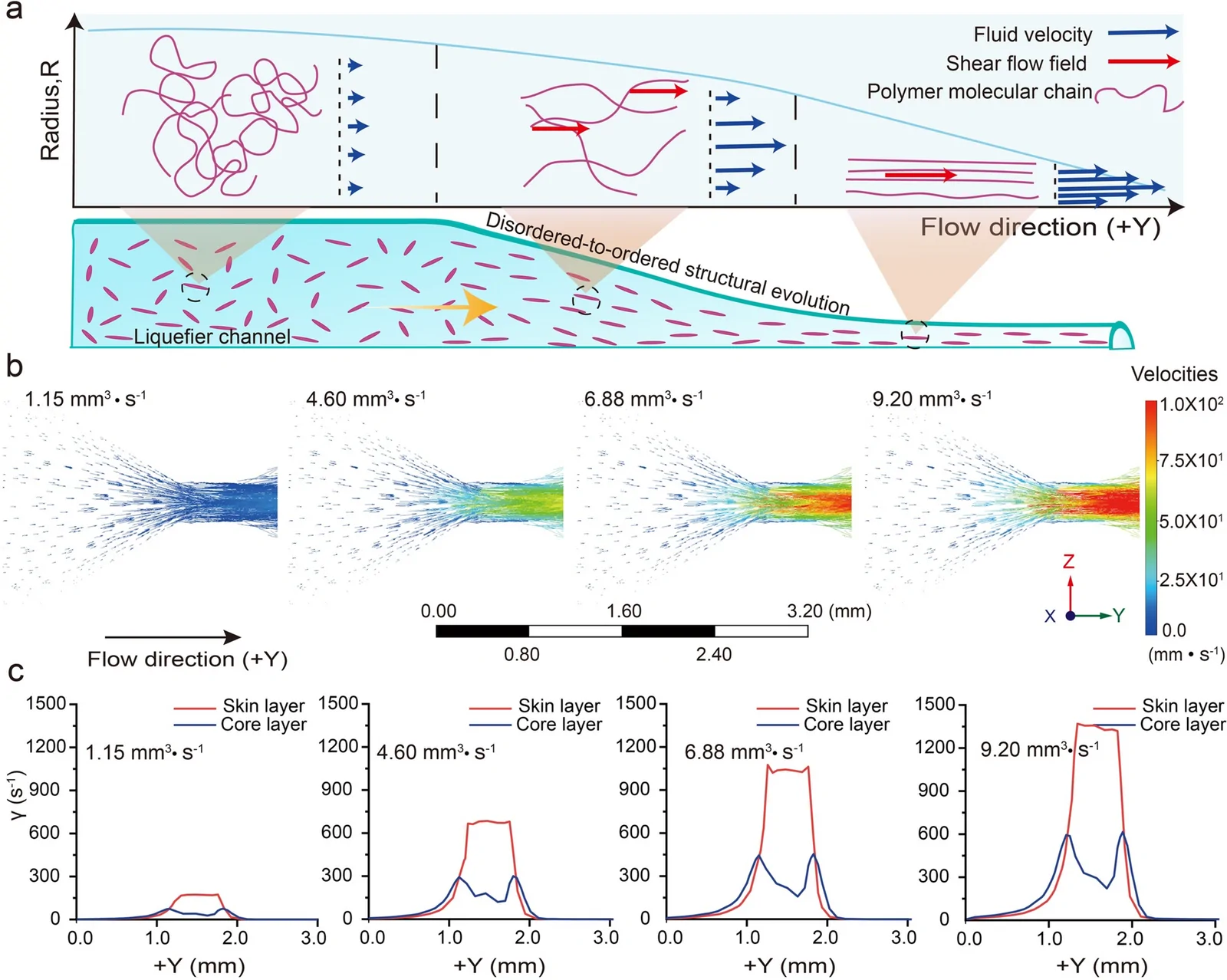
CFD simulation of fluids in a 3D printing flow field. **a** Schematic of the disordered-to-ordered structural evolution of molecular chains; **b** velocity vector distribution in convergence zone with different volume flow rates; **c** 1D-shear rate curves at skin and core layers

Different from the electric/magnetic field-driven strategies for customizing anisotropic microstructures, FDM 3D printing technology featuring a monodirectional shear flow field introduces high-freedom and universality for tailoring the micro-scale ordered structures of molecular chains and nanoparticles [27–29]. Figure 1a schematizes the potential structural evolution of molecular chains driven by a shear flow field in a 3D printing liquefier channel. To gain insight into the field-dependence of the formed microstructures, the dynamic mechanism of the fluids is systematically explored through CFD simulation (‌ANSYS Polyflow 2022). The geometric model, meshes, and boundary conditions of the liquefier channel are detailed in the Supporting Information for the subsequent calculations (Figs. S1 and S2, Table S2). As a result, the two-dimensional velocity distribution in a symmetrical profile along the liquefier channel at a specific volume flow (4.60 mm^3^ s^−1^) is shown in Fig. S3. Apparently, the fluid velocity is fully developed along the flow direction (+ Y) in the tubular channel, simultaneously accompanied by a significant increase of velocity in the convergence zone. The specific velocity vector (ν) of fluids with different volume flow rates in the convergence zone is recorded (Fig. 1b). A positive contribution to velocity is demonstrated with the increase of the volume flow rate, and the existing velocity gradient ($\Delta \nu$) perpendicular to the flow direction is conducive to establishing the shear flow field, thereby driving the construction of anisotropic microstructures. Based on the formula of $\gamma =\Delta \nu /\Delta l$ [30], where Δl is the difference in displacement perpendicular to the flow direction (+ Y), the corresponding 2D patterns of shear rate (γ) distribution and the converted 1D-shear rate curves at skin and core layers are calculated (Figs. S4 and 1c). In the axial direction, γ programmatically increases with increasing volume flow rate, resulting in the range of 1.4–2.5 × 10^3^ s^−1^. Meanwhile, the remarkable γ gradient ($\Delta \gamma$) in the radial direction provides the internal driving force for the disordered-to-ordered structural evolution of molecular chains. Typically, when the volume flow rate increases to 9.20 mm^3^ s^−1^, the significant enhancement of $\Delta \gamma$ is reaching ~ 1.0 × 10^3^ s^−1^ from ~ 1.0 × 10^2^ s^−1^ at 1.15 mm^3^ s^−1^, which is conducive to straightening molecular chains. Noteworthily, there exists a critical shear stress (${\tau }_{c}$) for viscoelastic fluids in the liquefier channel. When τ is larger than the specific value ($\tau \ge {\tau }_{c}$), a relative tangential velocity between fluid boundary and solid surface occurs, defined as the boundary slip of fluids [31]. This behavior would bring a negative contribution on $\Delta \nu$, thereby weakening γ and restraining the ordered alignment of molecular chains and coupled nanoparticles. Hence, the current issue is how to exclude the effect of boundary slipping actions on viscoelastic fluids, thus obtaining the actual γ value for evaluating the alignment behavior of molecular chains and coupled nanoparticles.

**Fig. 2:**
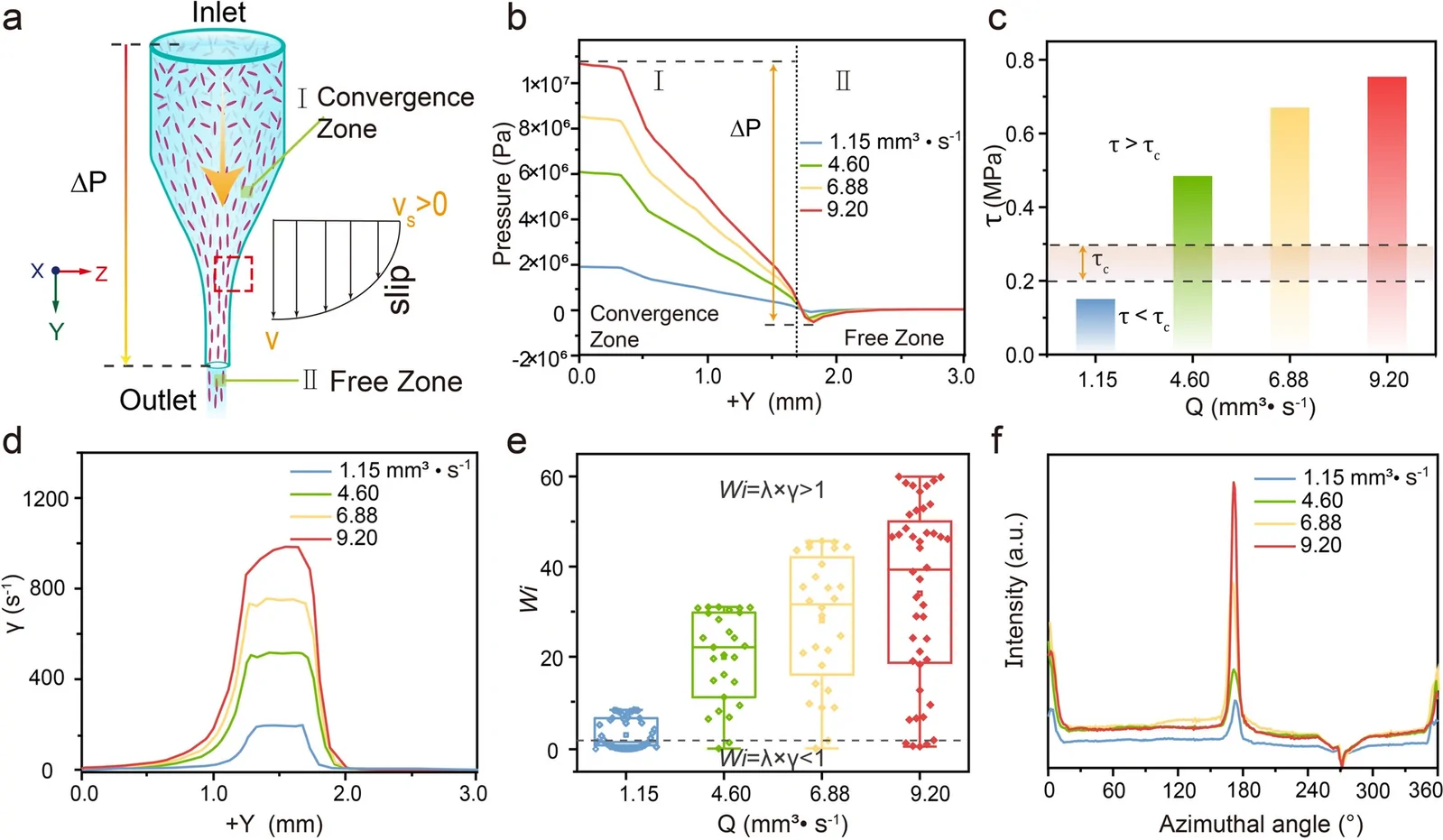
Dynamic mechanism of molecular chains in convergence zone. **a** Schematic of potential boundary slipping action of viscoelastic fluids; **b** 1D-pressure curves of fluids with different volume flow rates, and **c** the corresponding shear stresses; **d** modified 1D-shear rate curves at skin layer; **e** the calculated Weissenberg number; **f** 1D intensity-azimuthal angle curves of 3D-printed samples with different volume flow rates

Figure 2a depicts the schematic of potential boundary slipping action of viscoelastic fluids in the convergence zone during the 3D printing process. To eliminate the possible influence of boundary slip onto *γ*, *τ* in the convergence zone is calculated by the formula of $\tau =\frac{{R}\Delta P}{2{L}}$ [31], where R is the radius of channel, Δ*Ρ* is the pressure drop along flow direction, and L represents the length of the constrained channel. The 2D pressure distribution profiles of fluids with different volume flow rates and the converted 1D-pressure curves are displayed in Figs. S5 and 2b, respectively. With the continuous increase of volume flow rate, the Δ*Ρ* presents an increasing tendency, which implies the possibility of the *τ* of fluids breaking through the critical value (*τ*_*c*_) for activating boundary slipping action. Correspondingly, the *τ* values at different volume flow rates are further explored in Fig. 2c. Notedly, the critical shear stress (*τ*_*c*_) of polylactic acid fluids for boundary slip is in the range of 0.2–0.3 MPa [32]. In comparison, at a low volume flow rate of 1.15 mm^3^ s^−1^, $\tau <{\tau }_{c}$, the boundary slipping action is neglectable. Meanwhile, at other volume flow rates (4.60, 6.88, and 9.20 mm^3^ s^−1^), $\tau >{\tau }_{c}$ and boundary slip occurs. Thus, the modified 2D shear rate distribution patterns of fluids with different volume flow rates and the corresponding 1D-shear rate curves at the skin layer are described in Figs. S6 and 2d, respectively. A slight drop of the actual γ is revealed as compared the no-slipping condition (Fig. 1c). As an example, the maximum γ value at 9.20 mm^3^ s^−1^ decreases to ~ 1.0 × 10^3^ from ~ 6.1 × 10^2^ s^−1^ after boundary slipping.

After obtaining the actual γ, the Weissenberg number (Wi) is employed to quantitatively analyze the structural evolution of molecular chains and nanoparticles. It is expressed by the formula $\mathrm{Wi}=\gamma \times\lambda$ [33], where λ is corresponding to relaxation time, and is a dimensionless parameter reflecting the competitive relationship between the alignment of molecular chains by an external-field-driven action and the entanglement induced by its self-relaxation effect. Notably, as $\mathrm{Wi}>1$, the external-field driving action is dominant and the molecular chain alignment occurs; conversely, as $0<\mathrm{Wi}<1$, the self-relaxation effect plays a dominant role, and the molecular chains entangle, forming disordered structures [33]. Accordingly, the λ about 0.06 s of polylactic acid molecular chains at a reference temperature (190 °C) is fitted by a Bird–Carreau constitutive equation [34, 35], where a wide-frequency master curve is generated by the time–temperature superposition principle with various frequency sweeping curves at different measured temperatures (Fig. S7), to fit the relevant viscoelastic factors including infinite-shear-rate viscosity, zero-shear-rate viscosity, power-law index, and λ (Fig. S8). Thereafter, the $\mathrm{Wi}$ values in the convergence zone are successfully determined as shown in Fig. 2e. At the preset volume flow rates, the $\mathrm{Wi}$ values are greater than 1 ($\mathrm{Wi}>1$), implying a disordered-to-ordered tendency of chains in the liquefier channel driven by the shear flow field. In detail, at the fixed low volume flow rate of 1.15 mm^3^ s^−1^, the Wi values are distributed in the range of 1.2–8.1. Upon continuously increasing the volume flow rate, a visible increase of Wi is revealed, e.g., the average Wi increases to ~ 22 at the rate of 4.60 mm^3^ s^−1^, and a significant increase of Wi (~ 39) is apparent as the rate is raised to 9.20 mm^3^ s^−1^, implying a dominant role of the shear flow field onto the ordered alignment of molecular chains along the flow direction. To make a clear demonstration of the ordered alignment of molecular chains, a proof-of-concept experiment using the same preset procedure is carried out, and the resulted 3D-printed PLA samples with different volume flow rates are analyzed by 2D synchrotron small-angle X-ray scattering (SAXS). Their patterns are shown in Fig. S9. Consistently, all scattering patterns showed a strong “streak” signal on the equatorial line (parallel to the flow direction), which is attributed to the ordered alignment of molecular chains [36]. The converted 1D intensity-azimuthal angle curves are depicted in Fig. 2f. The typical peaks further verify the ordered structure of molecular chains, and meanwhile the peak intensity is significantly enhanced with the increasing volume flow rates, suggesting a positive relationship between the volume flow rate and the alignment of molecular chains, with the orientation degree (f) of the maximum sample as high as ~ 0.9. This disordered-to-ordered structural evolution has provided proof-of-concept for tailoring the micro-scale order of molecular chains and nanoparticles via a shear flow field in 3D printing liquefier channel.

### Micro-scale GNs Ordered Alignment Driven by Shear Flow Field

**Fig. 3:**
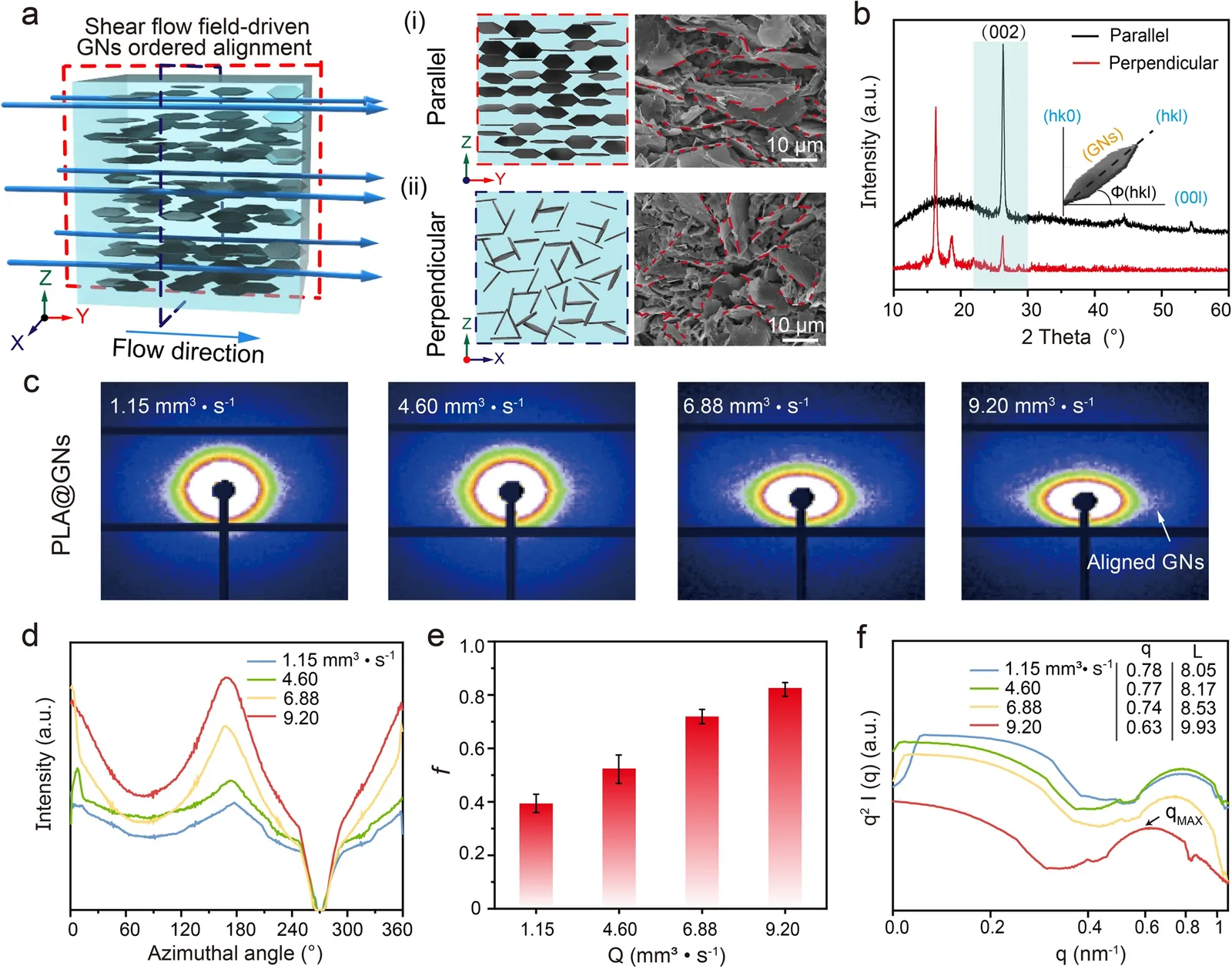
Morphologies of shear flow field-driven GNs ordered alignment. **a** Schematic of GNs ordered alignment and 2D profiles of GNs parallel/perpendicular to flow direction (i & ii); **b** XRD spectrum of GNs parallel/perpendicular to flow direction; **c** 2D-SAXS patterns of 3D-printed PLA@GNs samples with different volume flow rates, **d** the corresponding 1D intensity-azimuthal angle curves, **e** the orientation degree, and **f** the SAXS scattering intensity distribution curves

After fully analyzing the disordered-to-ordered structural evolution of molecule chains and nanoparticles in liquefier channel, we conduct the verified experiments regarding the micro-morphologies of PLA@GNs after 3D printing (Fig. 3). The schematic about the shear flow field-driven GNs ordered alignment and the corresponding 2D profiles parallel/perpendicular to the flow direction (i and ii) are depicted in Fig. 3a. Owing to the monodirectional shear flow field action, the two-degree-of-freedom GNs are directionally aligned in the long-range direction (i), but disordered in the perpendicular direction (ii) [37], which is simultaneously verified by SEM images of GNs parallel/perpendicular to the flow direction. Next, the XRD spectrum is used to assess the GNs ordered alignment in different directions (parallel/perpendicular to the field direction) (Fig. 3b). The inset presents a schematic illustrating the crystal plane of GNs, where *ϕ*(*hkl*) represents for the angle between the crystal plane (*hkl*) and the defined plane (00l), i.e., the characteristic peak (002) parallel to the defined plane is used to identify the ordered alignment of GNs [38, 39]. In comparison, the intensity of the characteristic peak (002) of the PLA@GNs sample parallel to the field direction is significantly higher than that of the sample perpendicular to the field direction, implying the successful implementation of GNs ordered alignment driven by the shear flow field in the 3D printing liquefier channel.

In more detail, the 2D-SAXS patterns of 3D-printed PLA@GNs samples preloaded with different volume flow rates are shown in Fig. 3c. All scattering patterns show a symmetrical “elliptic” signal on the equatorial line, mainly attributed to the aligned GNs along the field direction [40]. With the volume flow rate increasing, the eccentricity of patterns is increased in the equatorial direction, suggesting a positive contribution of shear action to the ordered alignment of GNs. To determine the f of GNs along the field direction, the converted 1D intensity-azimuthal angle curves are displayed in Fig. 3d. Accordingly, the f value of each sample is calculated by the full width at half maximum (FWHM) of the peaks in the intensity-azimuthal angle curves, by the following formula, *f* = (180° − FWHM)/180° [36]. As displayed in Fig. 3e, the positive impact of the rate on the f value is revealed, e.g., the f value increases from 0.39 for 1.15 mm^3^ s^−1^ to 0.53 for 4.6 mm^3^ s^−1^, and the maximum at 9.20 mm^3^ s^−1^ is 0.83. What is more, the corresponding SAXS scattering intensity distribution curves further confirm these conclusions (Fig. 3f). The long period (L) is calculated by the formula: L=2π/q [41], where q is the scattering vector of the peak. The obtained q value drops continuously, which in turn affects the increase of the L value, e.g., the q value decreased from 0.78 (1.15 mm^3^ s^−1^) to 0.63 (9.20 mm^3^ s^−1^), accompanied by the increase of L from 8.05 to 9.93 nm, suggesting an enhanced long-range ordered alignment of GNs along the flow field and an increased period distance with the increase of volume flow rate.

### Hierarchical Manufacturing of Anisotropic and High-Efficiency Shielding Modules

**Fig. 4:**
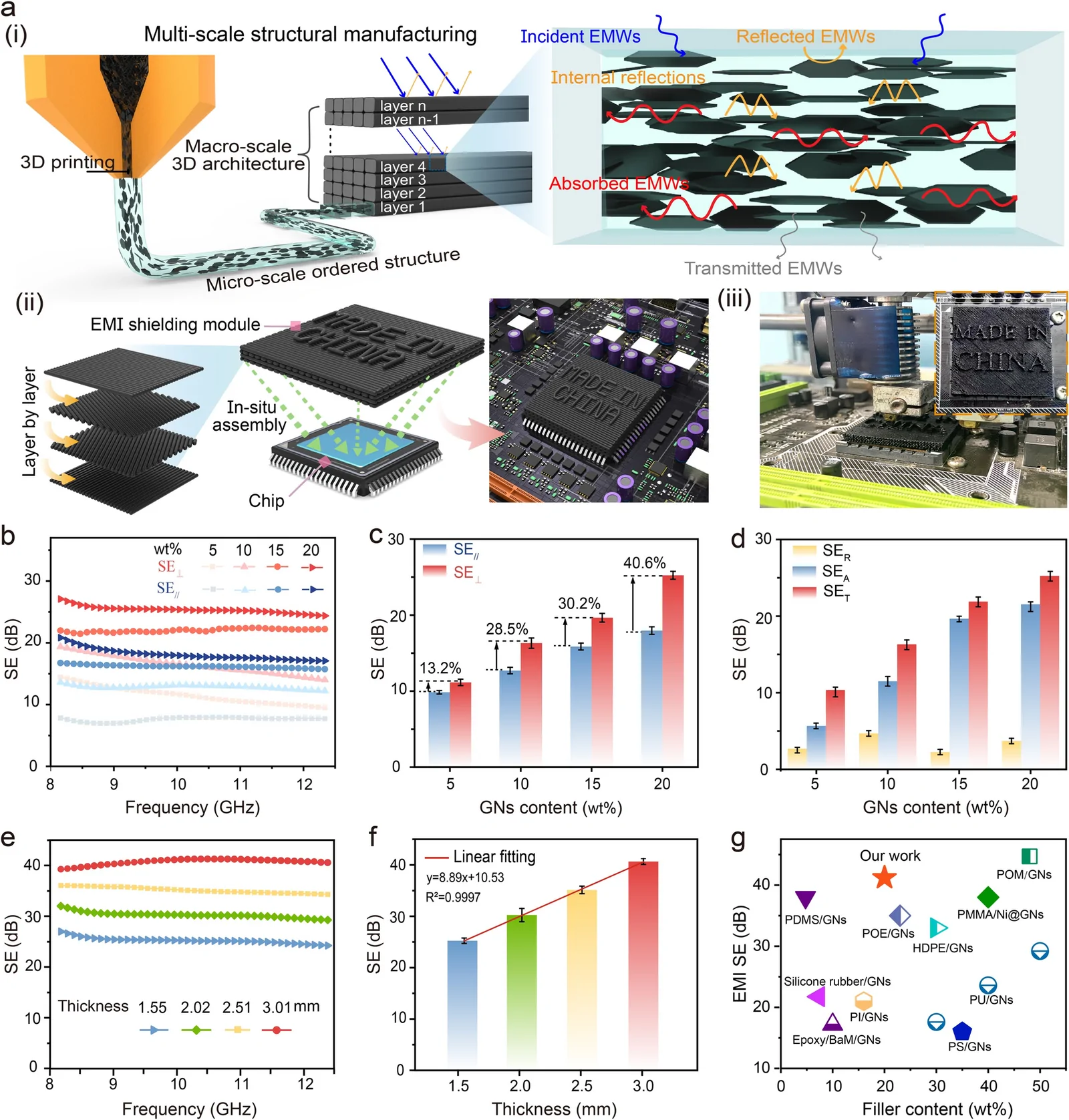
EMI shielding peculiarity of 3D-printed PLA@GNs materials. **a** Schematic of hierarchical manufacturing of PLA@GNs module; **b** EMI shielding properties parallel (SE_//_) or perpendicular to incident EMWs (SE_⊥_), **c** the average SE values, and **d** the corresponding electromagnetic parameters (SE_T_, SE_A_, and SE_R_) for the ordered structure perpendicular to incident EMWs; **e** EMI shielding properties as a variation of thickness and **f** the average SE values; **g** comparison of the shielding performance reported in this work and other previously reported GNs-based materials

Followingly, in order to fully leverage the synergistic effects of microscopic and macroscopic structures onto the comprehensive performance of shielding modules, a hierarchical manufacturing strategy is purposed by combining the precise control of GNs ordered alignment and the layer-by-layer assembly of PLA@GNs materials. Notedly, this hierarchically structural design would well utilize the macro/microstructural characteristics, thereby providing multilevel shielding for as-fabricated modules. The potential shielding mechanism is descripted in Fig. 4a that the sequentially arranged layers (e.g., layer 1 to n) act as barriers to attenuate EMWs, and meanwhile the absorptions and multiple internal reflections occur within a single layer, thereby dissipating the EMWs energy in the form of heat (i) [42, 43]. Besides, their potential EMC applications in electronic packaging and integration, and the shielding module in situ assembled onto the system on chip (SoC) are depicted in Fig. 3a(ii)–(iii) and S10. Accordingly, the scalable manufacturing of 3D-printed PLA@GNs shielding modules with different geometric sizes is also demonstrated in Fig. S11. Moreover, the intrinsic mechanical behaviors of PLA@GNs materials are also evaluated in Fig. S12. The maintaining Young's modulus of PLA@GNs material provides the desired stiffness to ensure the mechanical reliability of as-fabricated modules in their potential applications. Thereafter, along the preoptimized 3D printing trajectories (Fig. S13), the PLA@GNs samples with anisotropic structure are fabricated and their EMI shielding performances are measured (notedly, the shielding properties of samples are signed as SE_//_ and SE_⊥_, respectively, as the incident EMWs is parallel or perpendicular to the GNs ordered structure) (Fig. 4b). Overall, the SE_⊥_ and SE_//_ values have been significantly enhanced with the increasing GNs contents, which provides a fundamental guarantee for the EMC requirement of electronics in complex electromagnetic environments. Comparatively, the SE_⊥_ value is better than the SE_//_ value at the same GNs content, which is mainly due to the anisotropic electrical conductivity of sample by GNs ordered alignment (Fig. S14). The average SE values are further calculated for evaluating the contribution of the GNs ordered structure (Fig. 4c). A significant enhancement of SE_⊥_ is identified as compared to SE_//_, e.g., 13.2% increase for the PLA@GNs sample with 5 wt% GNs content, and 40.6% increase for the PLA@GNs sample with 20 wt% GNs content. Then, the shielding contributions of parameters including SE_T_, SE_A_, and SE_R_ of samples are also explored in Figs. S15 and 4d. Clearly, weather in parallel direction or perpendicular direction, SE_A_ plays a dominant role in the total shielding performance (SE_T_), which exhibits a similar trend with SE_T_ as increasing GNs contents, whereas SE_R_ is almost constant below 5 dB. Essentially, as the incident EMWs are perpendicular to the sample, the ordered GNs structures act as numbers of barriers efficiency that reflect and absorb EMWs energy within or between the sequentially arranged layers, thus realizing the expecting shielding performance [43, 44].

Moreover, the effect of thickness onto their shielding performance is investigated in Fig. 4e. The positive contribution of thickness on the samples’ shielding behavior is demonstrated. The average SE and the fitting curve are further displayed in Fig. 4f. Clearly, there is a linear correlation between the sample thickness and its SE value, and the maximum at a thickness of 3.01 mm is up to 41.2 dB, fully meeting the commercial EMI shielding standard (20 dB) for electronics [45]. Additionally, a comparison of the shielding performance of 3D-printed PLA@GNs and other previously reported GNs-based materials is provided in Fig. 4g and Table S3 that the PLA@GNs material reported in this work still demonstrates the expecting advantages in EMI performance over others. Thus, the scientific advances in this study are not only reflected in the excellent shielding performance of PLA@GNs materials endowed by the ordered structures of GNs, but also in the fact that the as-established universal methodology may be further extended to other polymer composite systems, thereby fully realizing its potential application value in various fields.

**Fig. 5:**
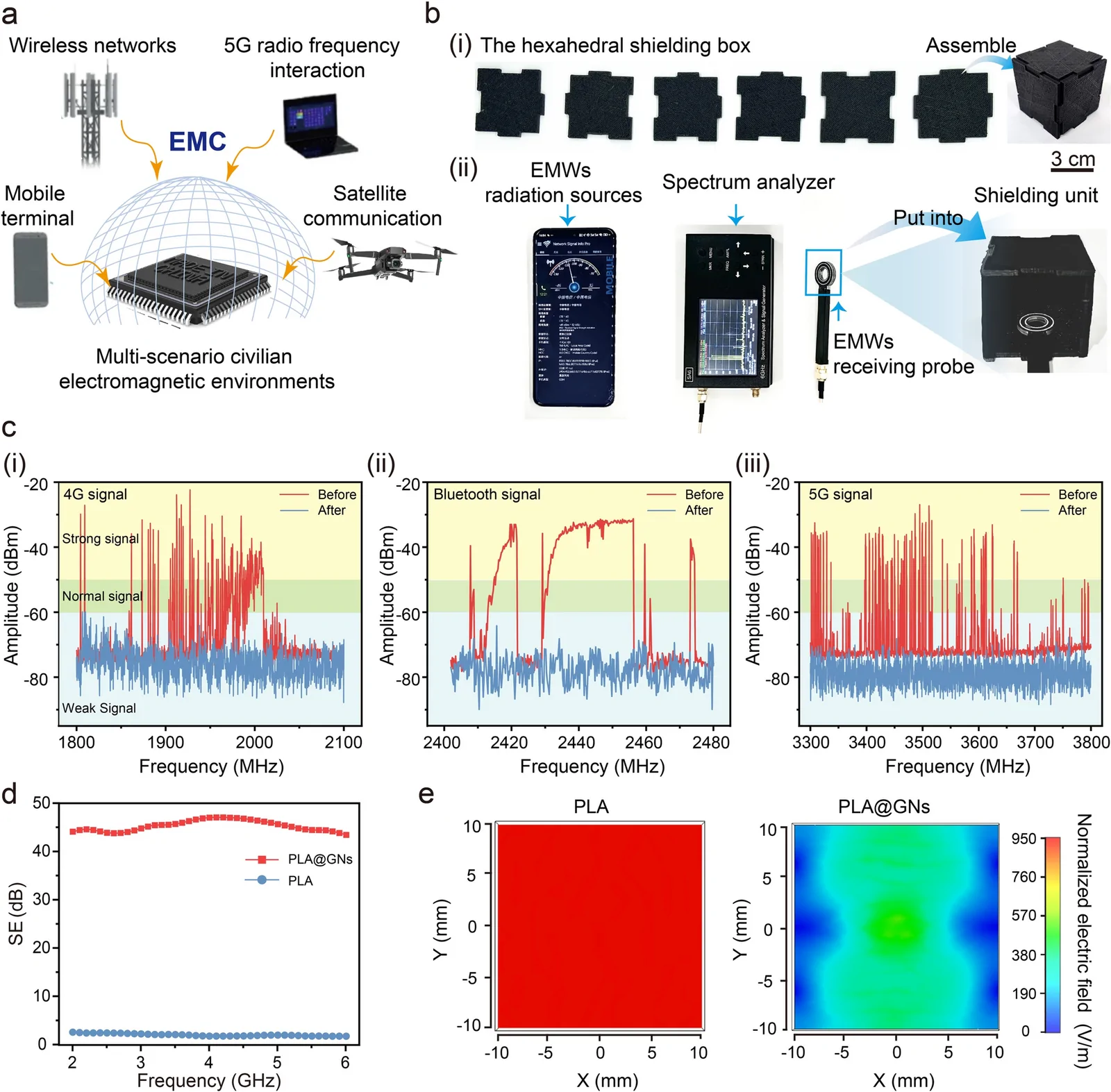
The potential shielding applications of 3D-printed PLA@GNs modules.** a** Schematic for EMI shielding in multiscenario civilian electromagnetic environments; **b** digital images of 3D-printed shielding box; **c** the real-time signal intensities in the 4G, Bluetooth, and 5G frequency bands before and after loading shielding box; **d** EMI shielding performance of PLA and PLA@GNs units in the civilian EMWs bands (2 ~ 6 GHz), and **e** the corresponding CST simulation

The promising shielding applications of 3D-printed PLA@GNs modules assembled onto core electronics for achieving the EMC in multiscenario civilian electromagnetic environments including mobile terminal, wireless networks, 5G radio frequency (RF) interactions, and satellite communication are investigated (Fig. 5a). As a proof-of-concept, a reliable verified experiment toward EMWs signals is conducted to assess the shielding potential of modules. It is accomplished by using a custom-designed shielding box and a portable EMWs signal detection system, including EMWs radiation sources, shielding units, and a spectrum analyzer equipped with a EMWs receiving probe (Fig. 5b). In detail, the hexahedral shielding box is assembled with the preprinted square shielding units (i), the specific civilian EMWs signals including 4G (1800–2100 MHz), Bluetooth (2402–2480 MHz), and 5G (3300–3800 MHz) frequency bands are simulated through a preinstalled mobile phone software, and the real-time signal intensities are monitored by a portable spectrum analyzer equipped with a *ϕ*23 mm ring-band metal probe (ii). The as-manufactured EMWs signal detection system is displayed in Fig. S16. Initial testing shows that as the signal intensity is within the range of − 100 ~ − 60 dBm, it is considered a weak signal source and difficult to detect by a metal probe. After an increase to − 60 ~ − 50 dBm, the normal signal could be detected; when the value is raised to − 50 ~ 0 dBm, a strong signal is measured by the metal probe [46]. Accordingly, the signal intensities at the 4G (1800–2100 MHz), Bluetooth (2402–2480 MHz), and 5G (3300–3800 MHz) frequencies are recorded before and after installing the shielding box (Fig. 5c). A similar trend is recorded in all frequency bands. Strong signal intensities within − 20 to 0 dBm, generated from the simulated signal sources, are effectively detected by the spectrum analyzer when the shielding box is removed, whereas these intensities drop to the range recorded for the weak signals of the − 60 ~ − 90 dBm range, when the metal probe is covered by the shielding box. Taking the 5G signal for explanation (iii), the max-hold signal intensity decreases from − 27 to − 77 dBm, demonstrating that the strong shielding of assembled box is applicable to the civilian signal bands.

Additionally, the corresponding EMI shielding property of pure PLA and PLA@GNs units is tested in the civilian EMWs bands (2–6 GHz) (Fig. 5d). Comparatively, the excellent shielding performance of PLA@GNs unit is demonstrated, where the maximum shielding value is as high as 45.2 dB, far exceeding the commercial shielding standard for EMWs (20 dB) [45, 47]. The electromagnetic simulation calculated by CST software is shown in Figs. S17 and 5e, which confirms the contribution of the shielding unit for EMWs. With the same test conditions, the PLA unit exhibits a high electric-field intensity, whereas the PLA@GNs unit results in a significant reduction of intensity, demonstrating the enhanced effectiveness of the shielding unit on protecting smart electronics from environmental electromagnetic signals [48].

### Thermal Management of 3D-Printed Functional Modules

**Fig. 6:**
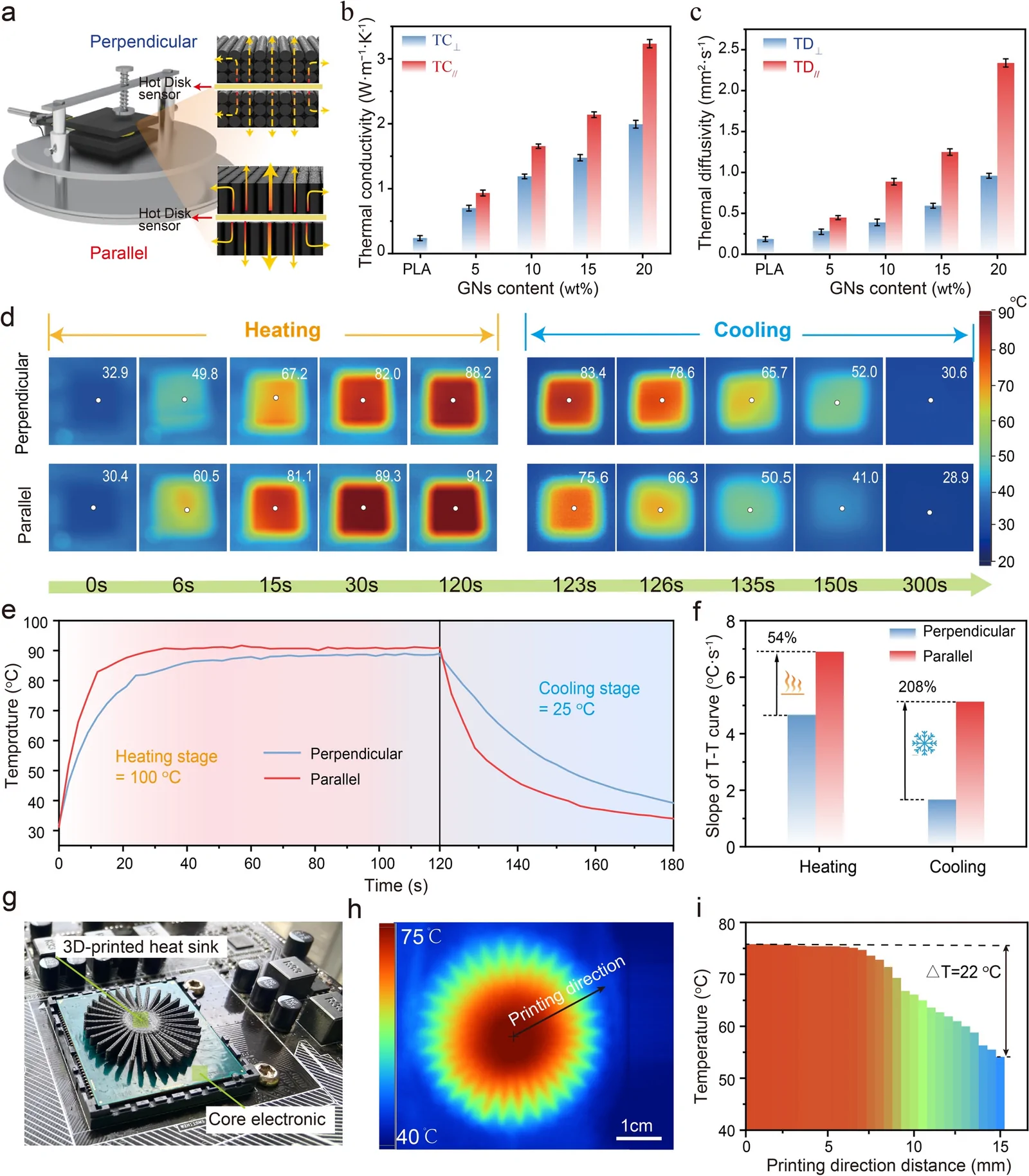
Thermal performance of 3D-printed PLA@GNs materials. **a** Schematic of heat-dissipation parallel or perpendicular to the ordered structure, **b** the thermal conductivity (TC_//_ and TC_⊥_), and **c** thermal diffusivity (TD_//_ and TD_⊥_); **d** the infrared thermal images of heat-dissipating behaviors parallel or perpendicular to ordered structure, and **e** the corresponding real-time heat-dissipation curves, as well as **f** the initial heating/cooling rates; **g** digital image of 3D-printed heat sinks, **h** the infrared thermal image of heat-dissipating behavior, and **i** the corresponding 1D-temperature curve along the ordered structure

Apart from the anisotropic and high-efficiency shielding property of 3D-printed modules, the anisotropic microstructure attributed to the GNs ordered alignment promotes the directional migration of electrons, thus strengthening the thermal conductivity in a specific direction [24, 49]. The thermal properties of 3D-printed PLA@GNs samples in different directions are assessed in Fig. 6. A schematic depicting the samples’ thermal conductivity (TC_//_ or TC_⊥_) and thermal diffusivity (TD_//_ or TD_⊥_) parallel or perpendicular to the GNs ordered structure is shown in Fig. 6a. Focusing on the thermal conductivity (Fig. 6b), a significant enhancement of TC value of PLA@GNs sample is recorded as compared to the pure PLA (0.23 W m^−1^ K^−1^). The TC_//_ value is much higher than the TC_⊥_ value at the same GNs content, which is attributed to the directional migration of electrons along the ordered GNs structures [50]. As the GNs content increases to 20 wt%, the TC_//_ value is as high as 3.2 W m^−1^ K^−1^, 68% higher than the TC_⊥_ (1.9 W m^−1^ K^−1^), and simultaneously ~ 13 times than that of the pure one. For the thermal diffusivity (Fig. 6c), a similar trend is recorded. The TD_//_ value of the PLA@GNs sample with 20 wt% GNs content is about to 2.3 mm^2^ s^−1^, 150% higher than the TD_⊥_ (1.0 mm^2^ s^−1^).

Moreover, the thermal-dissipation behaviors of samples in parallel or perpendicular direction are visually investigated under the controlled thermal environments (Fig. 6d), while the real-time temperature curves are synchronously recorded (Fig. 6e). It can be seen that the sample possesses a good thermal conduction/diffusion capability in the parallel direction, for which the maximum temperature difference is nearly 16 °C. This is mainly related to the highly-efficient thermal conduction of the GNs ordered alignment in the specific direction, resulting in TC_//_ > TC_⊥_. Meanwhile, the initial heating/cooling efficiency is fitted in Fig. 6f. In comparison, the heating/cooling efficiency in parallel direction achieves a 54% and 208% improvement compared to the perpendicular direction, respectively. These obtained results fully verify the positive contribution of the well-tailored GNs ordered structure to the anisotropic thermal conductivity of 3D-printed PLA@GNs samples [51, 52]. In order to take full advantage of the GNs ordered structure for the thermal dissipation of electronic components, a proof-of-concept involving a 3D-printed heat sink which is in situ assembled onto the core electronic is investigated, and the corresponding infrared thermal profile is displayed in Fig. 6g, h. In the thermal profile, the excellent thermal diffusion behavior is observed from the inside of the heat sink to the edge, i.e., the thermal energy is well dissipated along the aligned GNs network. Moreover, the 1D-temperature curve further confirms the contribution of the GNs ordered structure to the thermal dissipation of electronics, where the maximum temperature difference could reach ~ 22 °C (Fig. 6i). Overall, this innovative breakthrough on 3D-printed functional materials with well-designed GNs ordered networks paves the way for the electromagnetic compatibility and thermal management of smart electronics.

## Conclusions

In summary, this work proposes a hierarchical manufacturing strategy for fabricating PLA@GNs materials with anisotropic thermal conductivity and high-efficiency shielding against high-frequency/high-power electromagnetic waves by combining the shear flow field-driven GNs ordered alignment and layer-by-layer assembly. With the help of CFD simulations, the dynamic behavior of polymer fluids in 3D printing monodirectional flow fields is investigated and the disordered-to-ordered structural evolution of molecular chains is revealed. Additionally, the Weissenberg number (Wi) is employed to quantitatively evaluate the competitive relationship between the shear flow field-driven assembly and the thermal-motion driven relaxation of nanoparticles, thereby achieving the ordered alignment of molecular chains and GNs along the direction of the shear flow field. Subsequently, the 3D-printed PLA@GNs modules with anisotropic structural characteristic are assembled, and their potential contribution toward electromagnetic compatibility and heat dissipation of electronics is demonstrated. The 3D-printed PLA@GNs sample with an ordered GNs structure achieves an EMI shielding value of 41.2 dB in the X-band frequency range, far exceeding the commercial EMI shielding standard (20 dB). Moreover, the potential shielding application of the 3D-printed sample in specific civilian EMWs frequency bands such as 4G (1800–2100 MHz), Bluetooth (2402–2480 MHz), and 5G (3300–3800 MHz) is fully demonstrated. Besides, the directional thermal conductivity along the GNs ordered alignment is up to 3.2 W m^−1^ K^−1^, ~ 1300% higher than that of pure PLA, well suitable for the heat dissipation in a 3D-printed heat sink. From theoretical simulation to verified experiment, this study provides a novel methodology for assembling high-performance GNs-based electromagnetic functional materials, allowing to meet the urgent requirements for electromagnetic compatibility and thermal management of smart electronics.

## Supplementary Information

Below is the link to the electronic supplementary material.

Supplementary file1 (DOCX 8284 KB)
