# Supplementary material for: Hierarchical Manufacturing of Anisotropic and High-Efficiency Electromagnetic Interference Shielding Modules for Smart Electronics
Source: Nanomicro Lett. 2026 Jan 5;18:181. doi: 10.1007/s40820-025-02024-4 (PMC12765769; doi:10.1007/s40820-025-02024-4)
Supplement: Supplementary file 1 — Supplementary file1 (DOCX 8284 KB) [file 40820_2025_2024_MOESM1_ESM.docx]

Supporting Information for

**Hierarchical Manufacturing of Anisotropic and High-Efficiency Electromagnetic Interference Shielding Modules for Smart Electronics**

Shaohong Shi^1, 3, *^, Siwen Deng^1^, Yuheng Jiang^1^, Jiabin Chen^1^, Lukas Sporrer^2^, Fangchao Cheng^1, *^, Quanquan Guo^2, *^, Jingjing Jing^3^, Yinghong Chen^3, *^

^1^ Guangxi Key Laboratory of Processing for Non-ferrous Metals and Featured Materials, School of Resources, Environment and Materials, Guangxi University, No. 100, Daxuedong Road, Nanning 530004, People’s Republic of China

^2^ Max Planck Institute of Microstructure Physics, Weinberg 2, Halle (Saale), 06120, Germany

^3^ State Key Laboratory of Polymer Materials Engineering, Polymer Research Institute of Sichuan University, Sichuan University, No. 24 South Section 1, Yihuan Road, Chengdu 610065, People’s Republic of China

*Corresponding authors. E-mail: [shshichn@gxu.edu.cn](mailto:shshichn@gxu.edu.cn) (Shaohong Shi); [fangchaocheng@gxu.edu.cn](mailto:fangchaocheng@gxu.edu.cn) (Fangchao Cheng); [quanquan.guo@mpi-halle.mpg.de](mailto:quanquan.guo@mpi-halle.mpg.de) (Quanquan Guo); [johnchen@scu.edu.cn](mailto:johnchen@scu.edu.cn) (Yinghong Chen)

**S1 Experimental Part Related**

**S1.1 Experimental Section**

**Characterization**

A rotational rheometer (AR2000ex, TA Instruments, USA) was used to evaluate the rheological properties of PLA melts in small amplitude oscillation mode as a function of angular frequency (0.0625-625 rad·s^-1^) and fixed strain (1%) and gap (1.0 mm). Synchrotron small-angle X-ray scattering (SAXS) experiments were conducted at the BL16B1 beamline in the Shanghai Synchrotron Radiation Facility (SSRF) to investigate the structural changes of samples with different volume flow rates. The X-ray wavelength used was 0.124 nm, with sample-to-detector distances set at 2289 mm for SAXS measurements. Two-dimensional (2D) SAXS patterns were recorded in transmission mode with exposure times of 5 s.

The surface morphology of the materials with two different printing orientations was measured using a field emission scanning electron microscope (SEM) (SU8020, Hitachi, Japan) with an accelerating voltage of 20 kV. The orientation of GNs in the printed parts in different printing modes with a size of 10×10×1 mm^3^ was investigated using an x-ray diffractometer (XRD, Bruker, Germany). The scanning angle (2θ) ranged from 10° to 60° and the scanning rate was set to 5°·min^-1^.

The electrical conductivity of the samples was assessed by measuring their volume resistance using a multifunctional digital multimeter (B41T+, Fujian Liliupu Optoelectronics Technology Co., Ltd., China). The intensity of the specific civilian electromagnetic pulse signal simulated by the mobile phone software was measured using a spectrum analyzer (SA6, Shenzhen Guoshang Technology Co., Ltd., China). The EMI SE characteristics of PLA@GNs nanocomposite samples with a diameter of 13.0 mm and different thicknesses printed in two different modes were recorded at 8.2-12.4 GHz and 2.0-6.0 GHz respectively using a vector network analyzer (Agilent N5230, USA). The electromagnetic parameters (SE_total_, SE_A_, SE_R_) and EMWs transmittance were calculated based on the factors (S_11_, S_12_, S_22_, S_21_), and the shielding parameters were all in accordance with the following equations [S1, S2]:

|  | $R=\left\vert S_{11} \right\vert^{2}=\left\vert S_{22} \right\vert^{2}$; $T=\left\vert S_{12} \right\vert^{2}=\left\vert S_{21} \right\vert^{2}$ | (S1) |
| --- | --- | --- |
|  | $A=1-R-T$ | (S2) |
|  | $\mathrm{SE}_{A}=-10\log\left( T/(1-R) \right)$ | (S3) |
|  | $\mathrm{SE}_{R}=-10\log\left( 1-R \right)$ | (S4) |
|  | $\mathrm{SE}_{\mathrm{total}}=\mathrm{SE}_{R}+\mathrm{SE}_{A}+\mathrm{SE}_{m}$ | (S5) |
|  | $=\sqrt{1/\pi f\sigma\mu}$ | (S6) |

where, $R$ is the reflection coefficient, T is the transmission coefficient, A is the absorption coefficient, SE_A_ is the absorption of electromagnetic waves, SE_R_ is the reflection of electromagnetic waves, SE_total_ (or EMI SE) is the total shielding property of sample, and SE_m_ is the multiple internal reflection of electromagnetic waves. f represents frequency; σ and μ represents electrical conductivity and permeability, respectively. Noticeably, SE_total_ > 10 dB, SE_m_ could be neglected [S3].

The thermal conductivity (TC) and thermal diffusivity (TD) of the samples with square structure (20 × 20 × 2 mm^3^) printed in different modes were tested using a thermal analyzer (Hot Disk 2500-OT, Sweden) and a laser scintillator (LFA467, NEXTZSCH, Germany). The samples with dimensions of 20 × 20 × 2 mm^3^ were placed on a heated platform with the platform temperature set to 100 °C at this constant temperature, and then quickly moved to a cold steel plate and cooled naturally in the air. An infrared thermal camera (FLIRONE Pro, FLIR, USA) was placed above it for real-time temperature measurement of the materials.

**S1.2 Finite Element Simulation**

Computational fluid dynamics (CFD) of PLA melts during FDM 3D printing were conducted by using ANSYS POLYFLOW software with an ICEM module for meshing the structure of 3D printing liquefier channel. The key equations including the continuity equation, the momentum equation, and the constitutive equation, are presented here for the Cartesian coordinate system [S4].

|  | $\nabla\cdot\nu=0$ | (S7) |
| --- | --- | --- |
|  | $-\nabla p+\nabla\cdot\tau=0$ | (S8) |
|  | $\tau=2\eta\left( \gamma\right)D$ | (S9) |
|  | $=\sqrt{1/\pi f\sigma\mu}$ | (S10) |

In these equations, $\nu$ represents the velocity vector, $p$ is the pressure, $\tau$ is the stress tensor associated with the viscous fluid, $\eta$ stands for the apparent viscosity, $\gamma$ is the shear rate, and $D$ is the tensor for deformation. The Brid-Carrera viscosity model for polymer PLA melt materials, taking into account the effects of temperature, shear rate, and pressure on the flow characteristics, can be described as follows [S5, S6]:

|  | $\eta=\eta_{\infty}+( \eta_{0}-\eta_{\infty})\left[ {1+( \lambda\gamma)}^{a} \right]^{\frac{n-1}{a}}$ | (S11) |
| --- | --- | --- |

where, $\eta_{\infty}$ is the infinite-shear viscosity (general infinite-shear viscosity is equal to 0), $\eta_{0}$ is the zero-shear viscosity, λ is the relaxation time, The Brid-Carrear viscosity model a is the shift factor of 2, $n$ is the power-law index. A rotational rheometer was used to investigate the rheological behavior of the melt under different oscillatory strain scanning modes, and the flow parameters of the PLA melt were fitted. The boundary conditions of the melt in the flow channel simulation are assumed as follows: (1) constant volumetric flow from the inlet to the outlet boundary; (2) at the planes, a zero traction was assumed; and (3) at the wall, the slip velocity is set as 10^6^ and $+\infty$, respectively.

Nevertheless, it is a verifiable fact that wall slip and viscous dissipation under channel scale conditions interact with each other and ultimately affect the flow characteristics of the fluid in the microfluidic channel [S7]. The Navier model, based on the adsorption-desorption mechanism and incorporating the generalized Navier's law, has been widely used in the microspray melt flow problem, as follows [S8]:

|  | $\tau_{w}=k(\nu_{w}-\nu_{s})\left\vert\nu_{s}-\nu_{w} \right\vert^{e-1}$ | (S12) |
| --- | --- | --- |

where, $\tau_{w}$ is the shear stress of the melt at the wall; $k$ is the slip factor; $\nu_{w}$ is the tangential velocity at the wall; $\nu_{s}$ is the velocity of the melt at the wall; e is the material parameter, which is usually taken to be the melt power-law index. Since the nozzle is usually stationary in the direction of the melt flow rate during extrusion, i.e., $\nu_{w}=0$, equation (12) is rewritten as [S9]:

|  | $\tau_{w}=-k{\nu_{s}}^{e}$ | (S13) |
| --- | --- | --- |

it should be noted that a slip factor of k indicates a greater degree of slip; when $k=0$, the wall slips completely; when $k=\infty$, the wall has no slip.

CST simulations were performed to analyze the electric field distribution within the composite materials, using a model with dimensions of 20 × 20 × 2 mm^3^. The dielectric constant and magnetic permeability of PLA and PLA@GNs samples were obtained by using a vector network analyzer.

**Table S1** The composition of PLA@GNs samples with different filler loading.

| GNs loading | GNs (g) | PLA (g) |
| --- | --- | --- |
| 5 wt% | 2.5 | 50 |
| 10 wt% | 5 |  |
| 15 wt% | 7.5 |  |
| 20 wt% | 10 |  |

**S2 Results & Discussion Related**


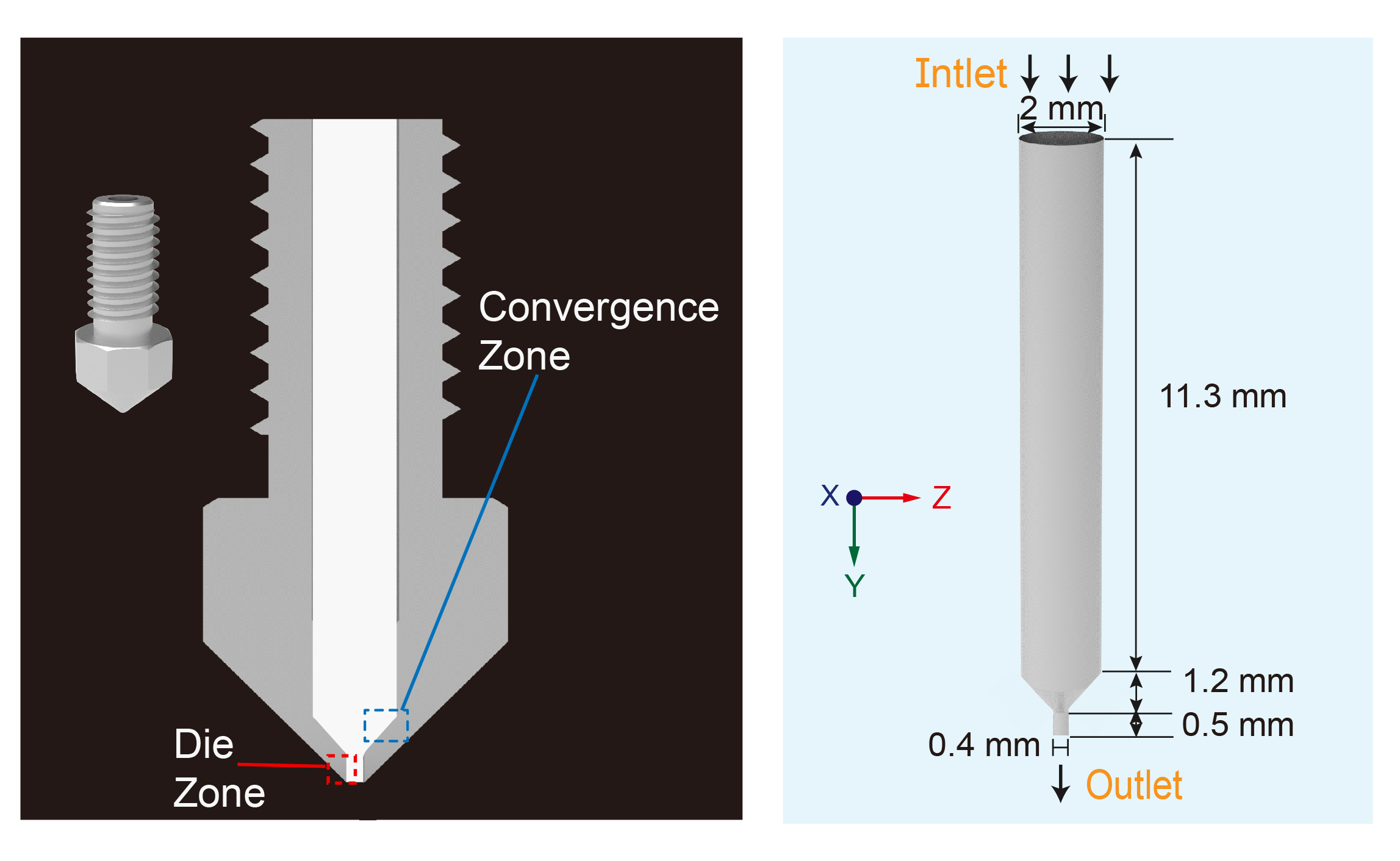


**Fig. S1** 3D printing nozzle and the corresponding geometrical model


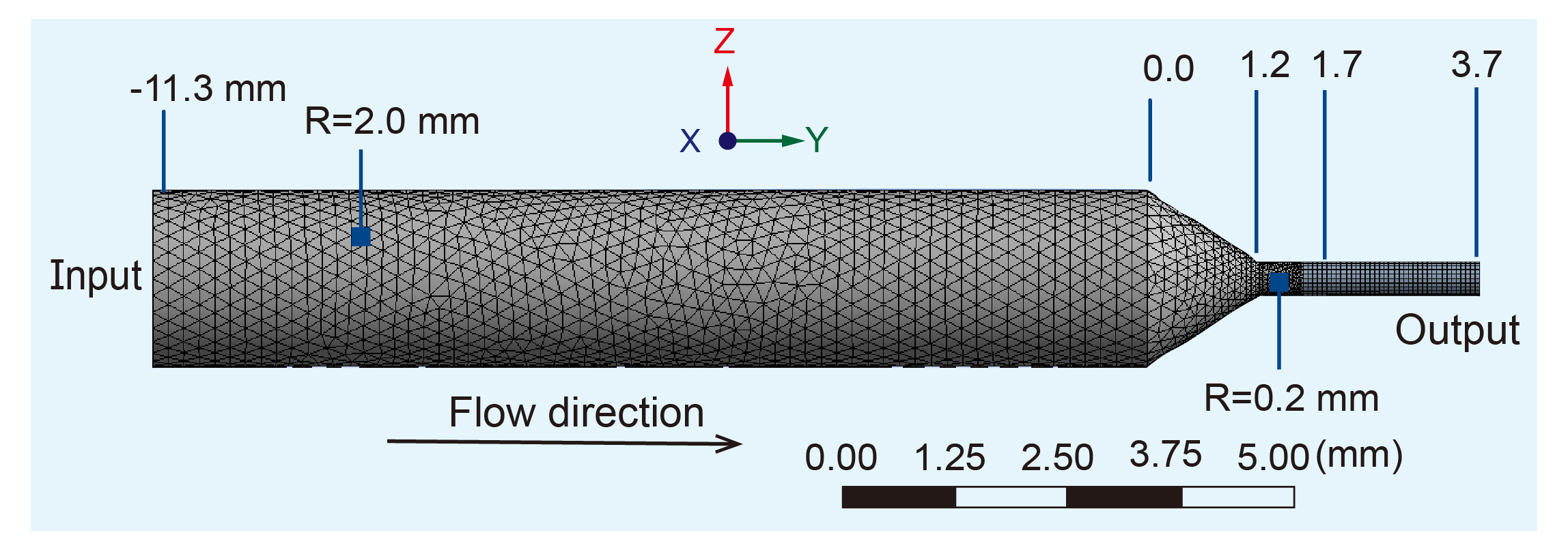


**Fig. S2** Mesh of 3D printing liquefier channel

**Table S2** The simulation parameters used for boundary slip behaviors

| Simulation parameters | Set values | | | |
| --- | --- | --- | --- | --- |
| Volume flow rate | 1.15 mm^3^·s^-1^ | 4.60 mm^3^·s^-1^ | 6.88 mm^3^·s^-1^ | 9.20 mm^3^·s^-1^ |
| Infinite-shear viscosity | 0 Pa·s | | | |
| Zero-shear viscosity | 1861 Pa·s | | | |
| Relaxation time | 0.06 s | | | |
| Power-law index | 0.84 | | | |
| Boundary Conditions | X/Y: unit cell, Z: open | | | |
| Slip condition | k: 10^6^, e: 0.84 | | | |


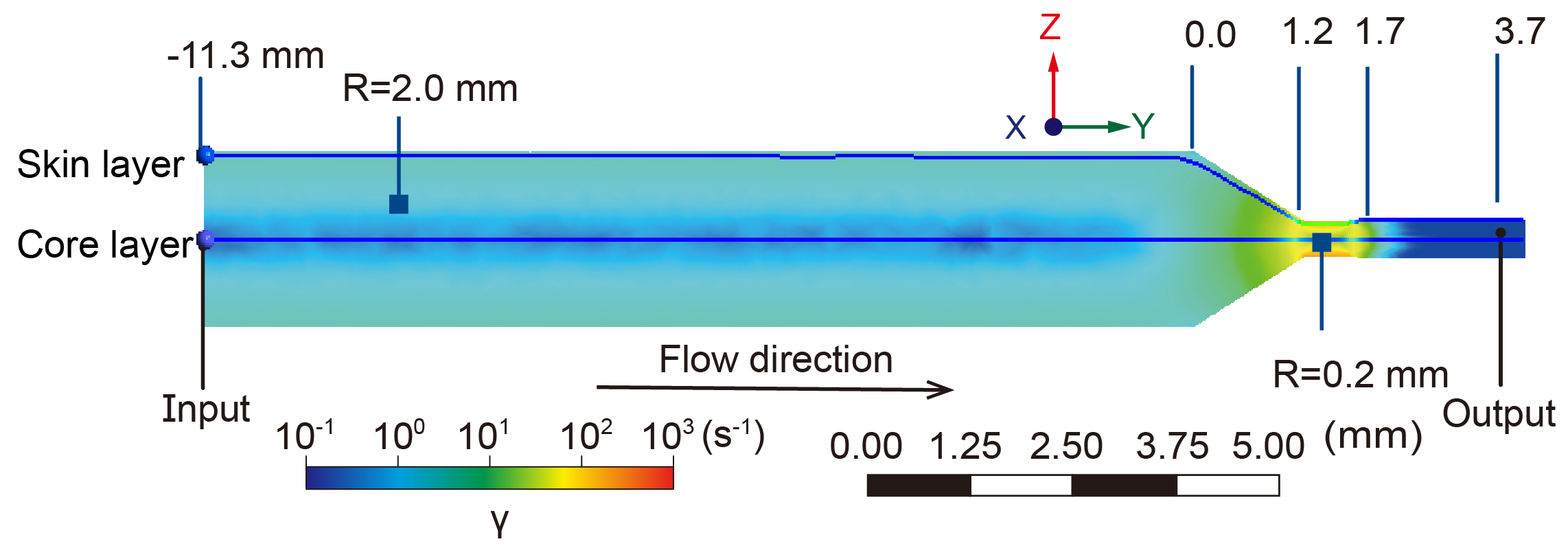


**Fig. S3** Two-dimensional velocity distribution in a symmetrical profile along the liquefier channel at a specific volume flow (4.60 mm^3^·s^-1^)


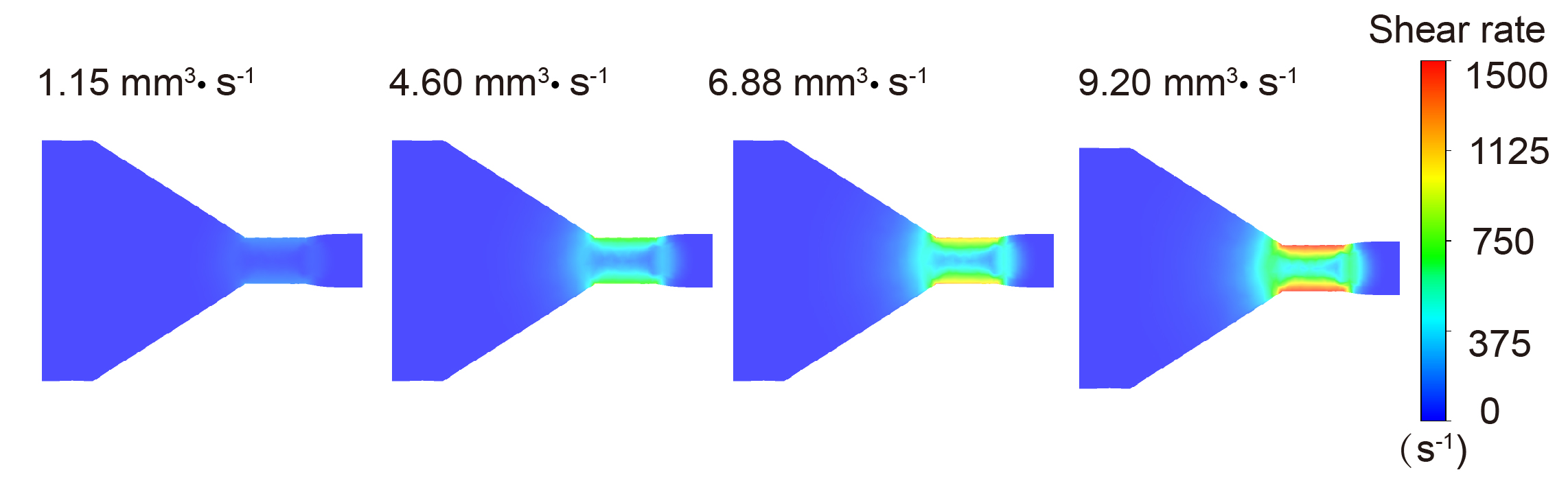


**Fig. S4** 2D patterns of shear rate ($\gamma$) distribution of fluids in convergence zone with different volume flow rates


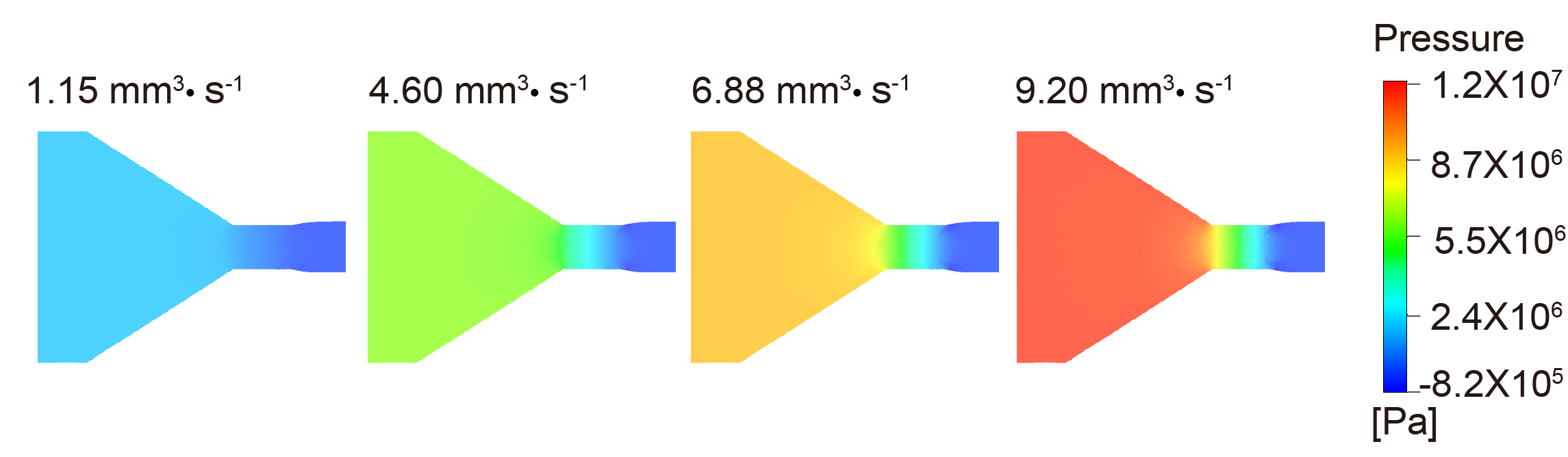


**Fig. S5** 2D pressure distribution of fluids in convergence zone with different volume flow rates


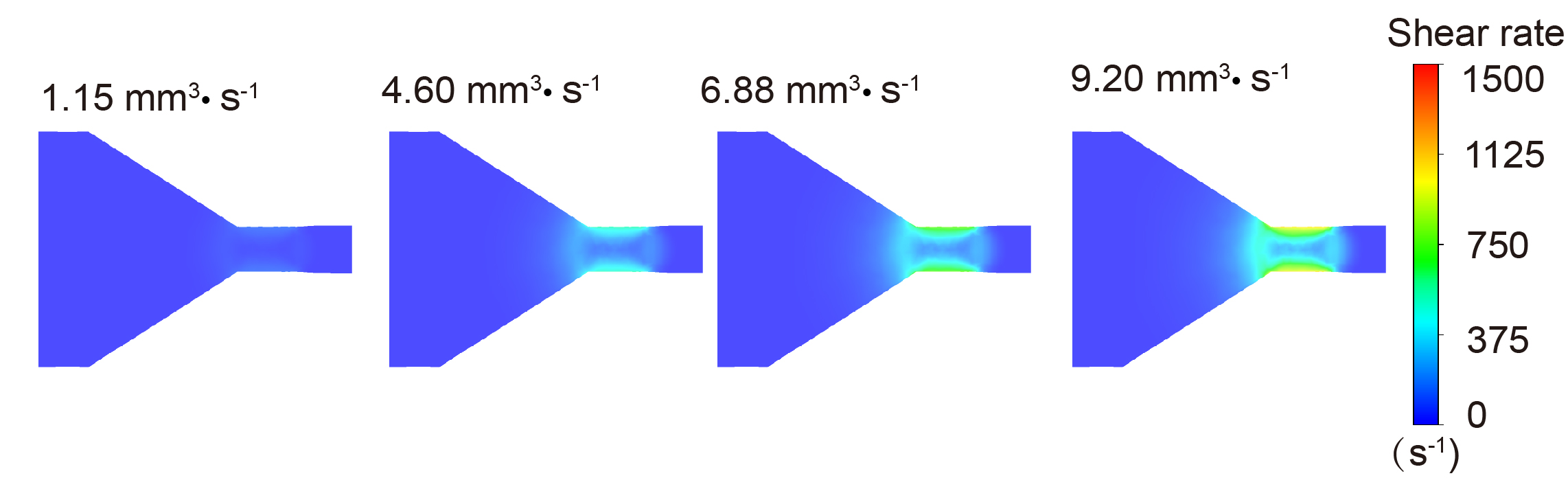


**Fig. S6** The modified 2D shear rate distribution of fluids with different volume flow rates


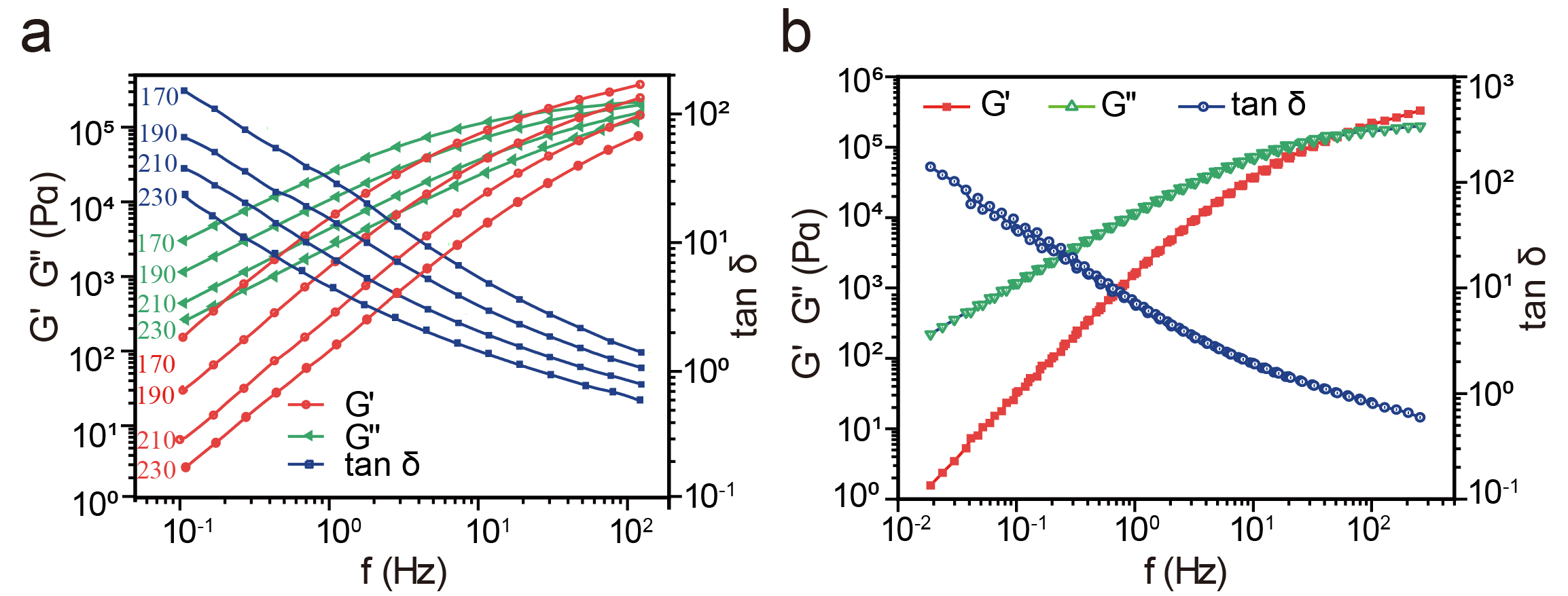


**Fig. S7 a** Storage module, loss module and loss tangent *vs.* frequency curves of pure PLA fluids at different temperatures; **b** The wide-frequency master curve at a reference temperature of 190 °C by time-temperature superposition principle


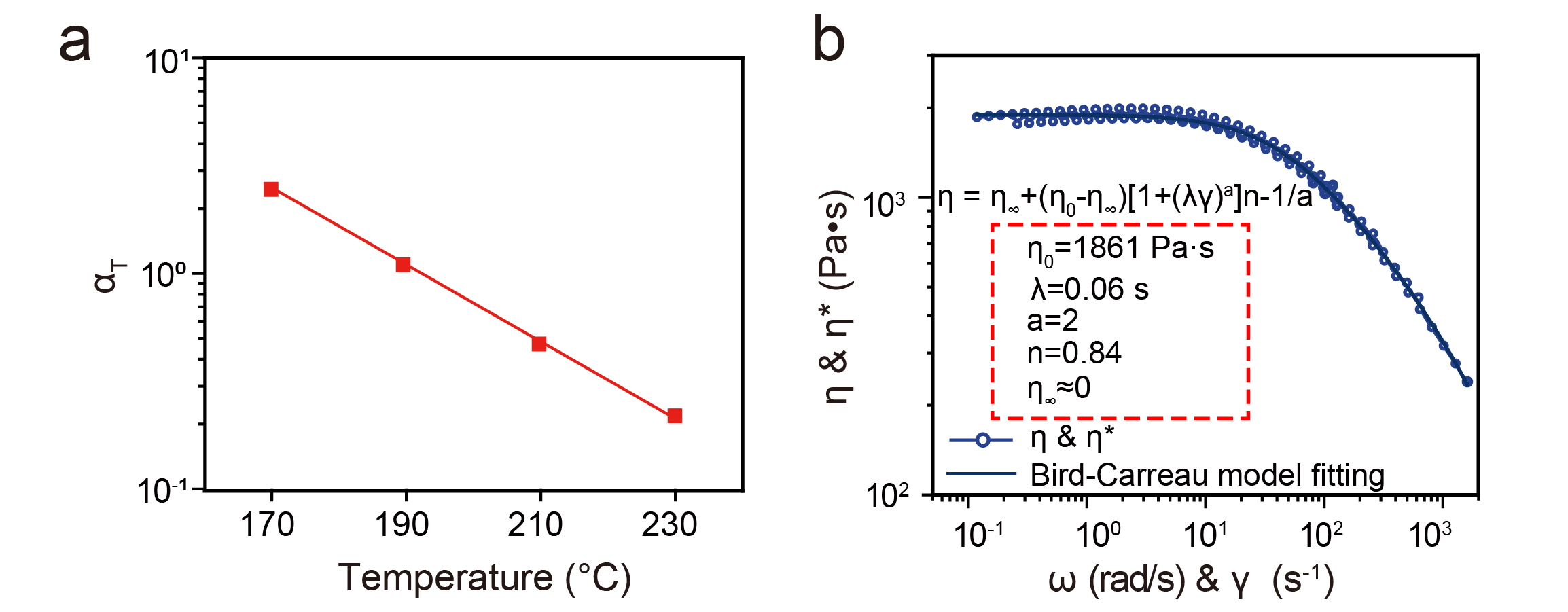


**Fig. S8 a** The shift factors at a reference temperature of 190 °C by time-temperature superposition principle; **b** The fitting viscoelastic parameters including infinite-shear-rate viscosity (η_∞_), zero-shear-rate viscosity (η_0_), relaxation time (λ) and power-law index (n)


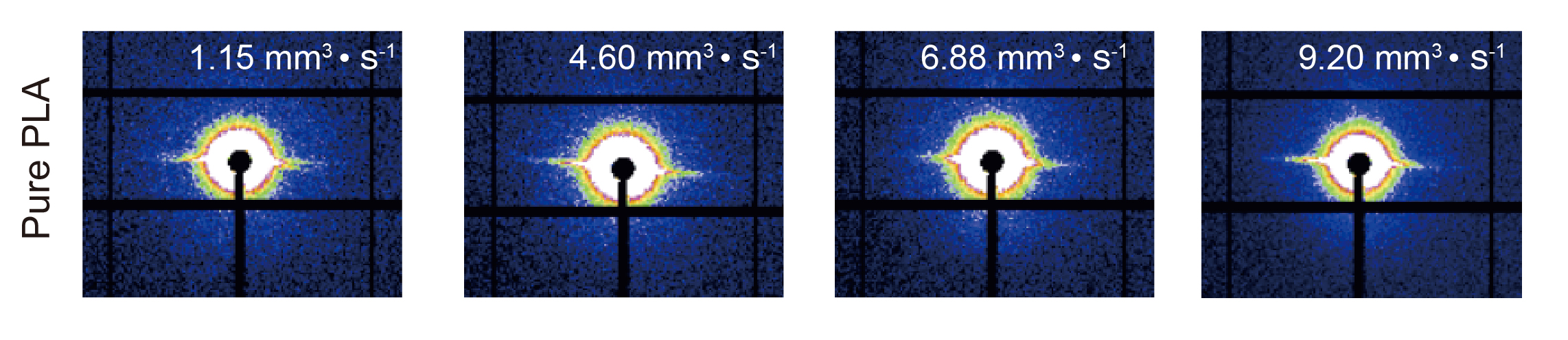


**Fig. S9** 2D synchrotron small-angle X-ray scattering (SAXS) patterns of 3D-printed PLA samples with different volume flow rates


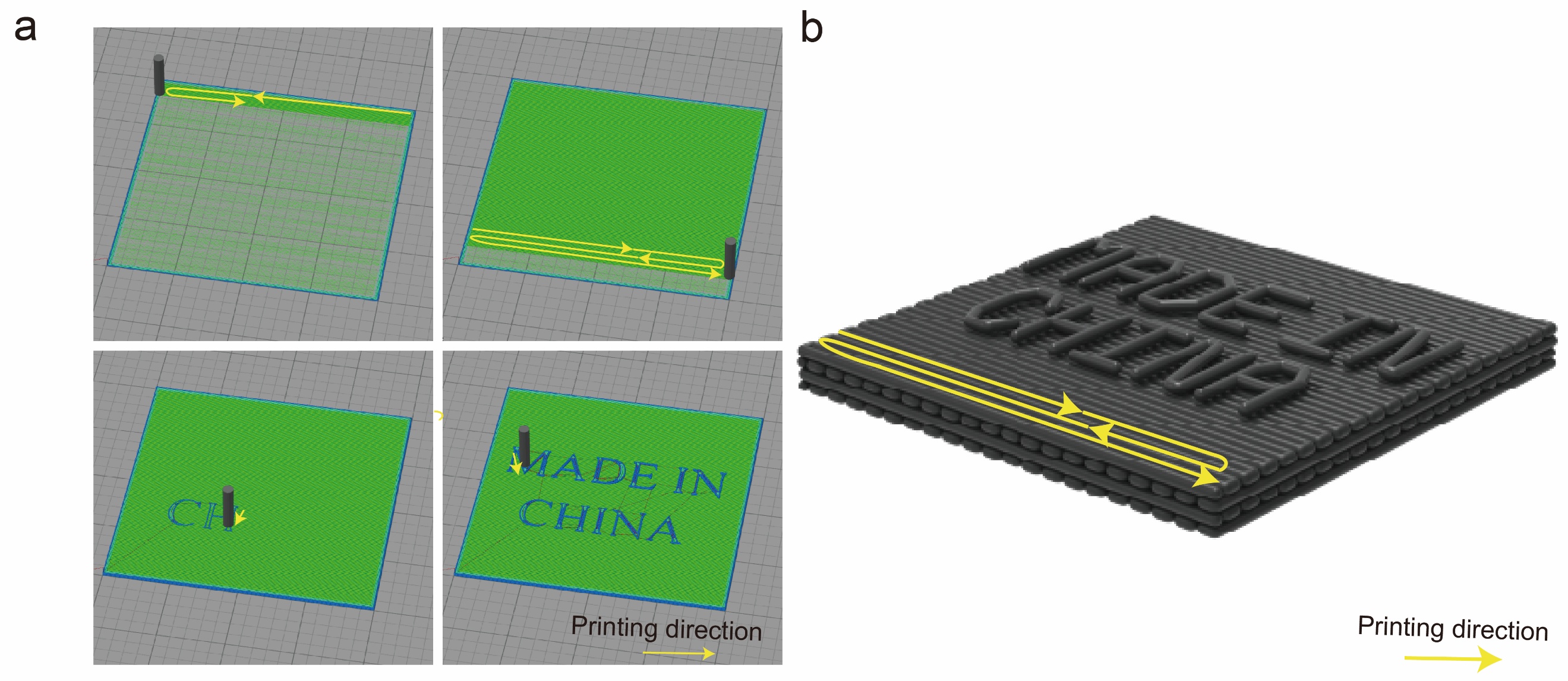


**Fig. S10** **a** Illustration of the 3D printing paths for shielding module; **b** The designed 3D printed shielding module.


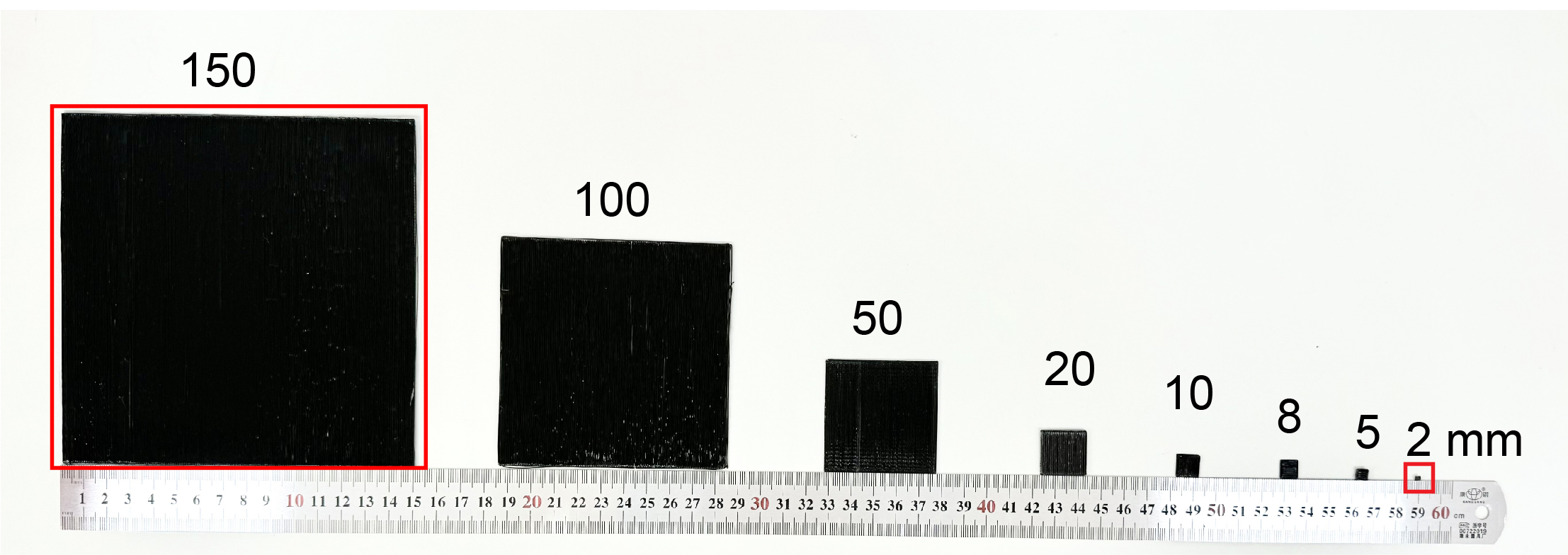


**Fig. S11** Scalable manufacturing of 3D-printed PLA@GNs shielding modules with different geometric sizes


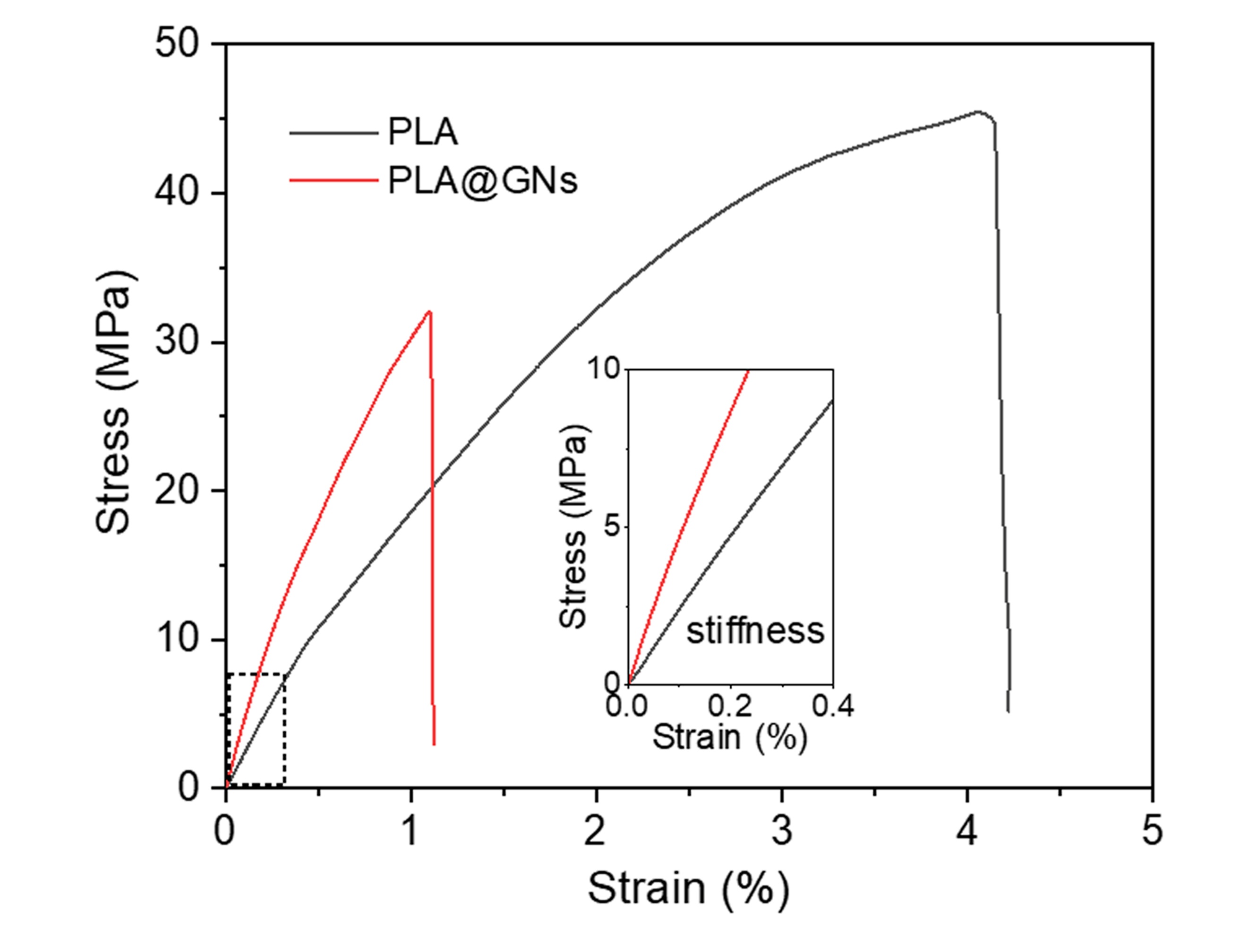


**Fig. S12** The representative stress-strain curves of pristine PLA and PLA@GNs composite with 20 wt.% GNs content


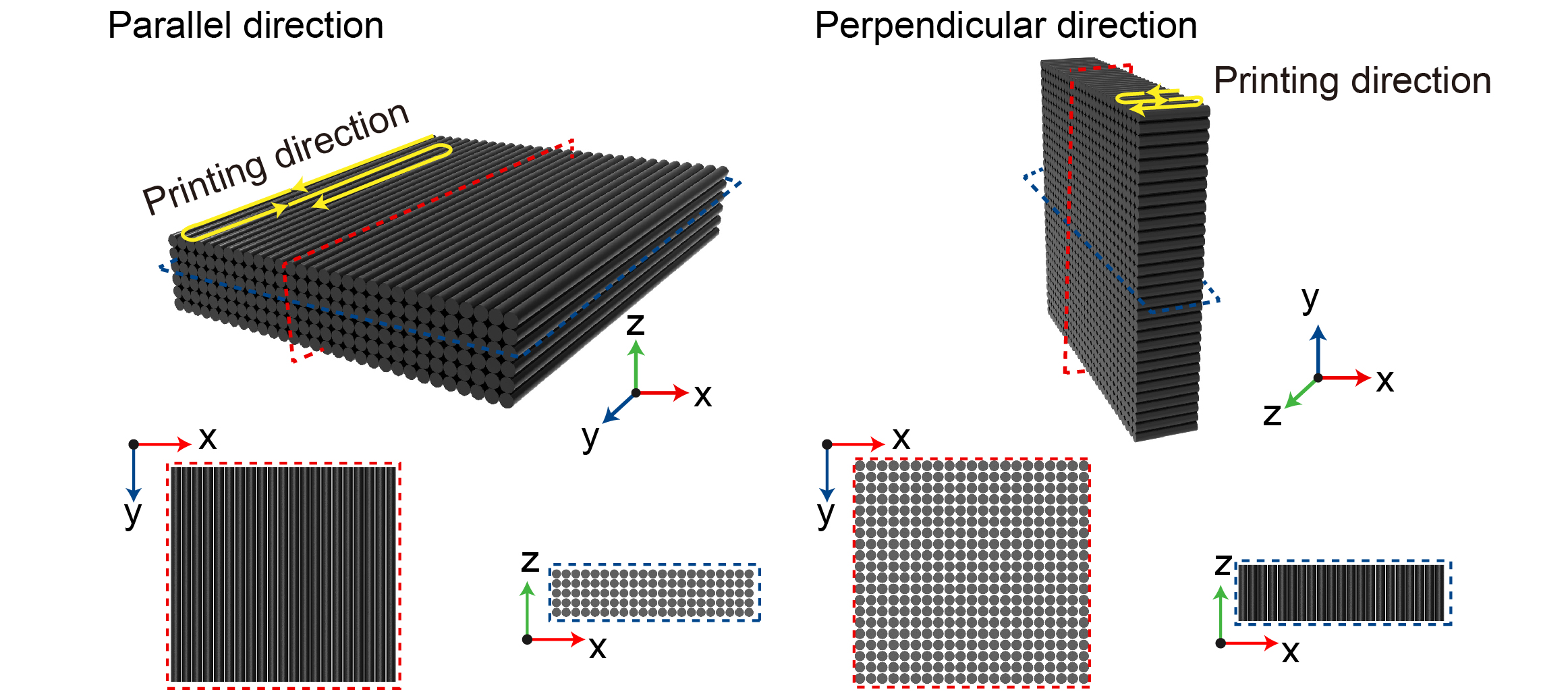


**Fig. S13** The characteristic 3D printing routes of samples with parallel or perpendicular alignments in three dimensional coordinates


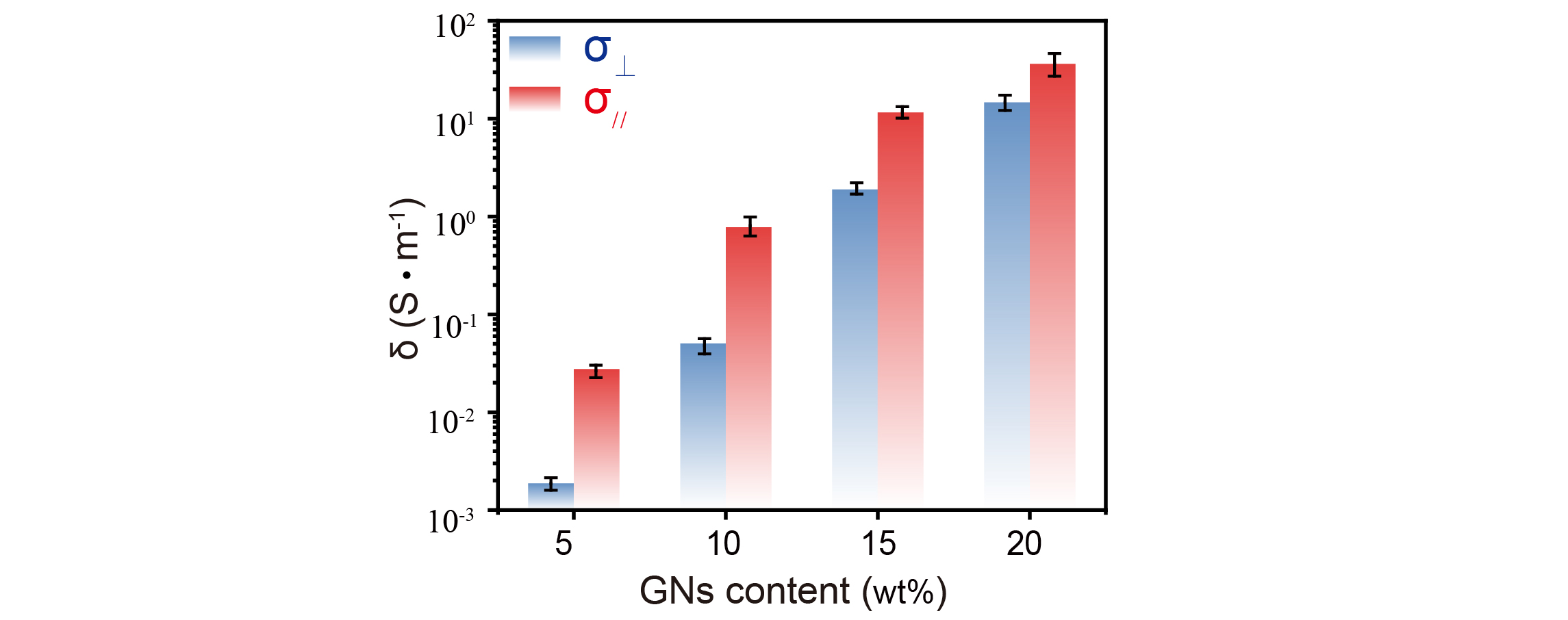


**Fig. S14** The anisotropic electrical conductivity of samples parallel to the ordered structure (σ_//_), and perpendicular to the ordered structure (σ_⊥_)


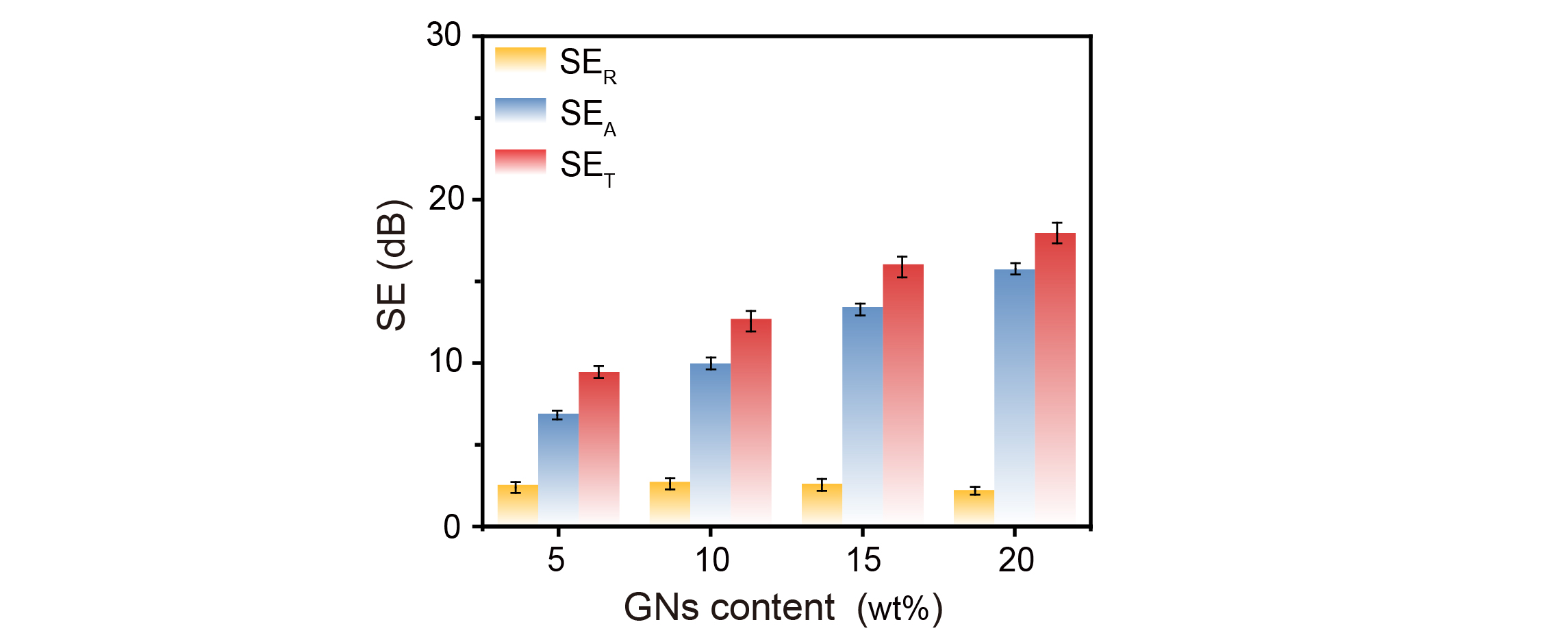


**Fig. S15** Electromagnetic parameters (SE_T_, SE_A_, and SE_R_) of samples with the ordered structure parallel to incident EMWs

**Table S3** Comparison on the shielding performance contributed by 3D-printed PLA@GNs sample and other previously reported GNs-based materials fabricated by traditional manufacturing techniques.

| **Composites** | **Manufacturing** | **EMI SE (dB)** | **Filler content (wt.%)** | **Refs.** |
| --- | --- | --- | --- | --- |
| PU/GNs | Polymer-infiltrated technique strategy | 29.2 | 50 | [S10] |
| PU/GNs | Polymer-infiltrated technique strategy | 23.6 | 40 | [S10] |
| PU/GNs | Polymer-infiltrated technique strategy | 17.6 | 30 | [S10] |
| PMMA/Ni@GNs | Solution blending | 38 | 40 | [S11] |
| Silicone rubber/GNs | Compression molding | 21.75 | 7.29 | [S12] |
| PI/GNs | Nonsolvent induced phase separation | 21 | 16 | [S13] |
| PS/GNs | Melt processing-hot pressing | 16 | 35 | [S14] |
| HDPE/GNs | Hot compression molding | 33 | 30 | [S15] |
| PDMS/GNs | Vacuum impregnation | 38 | 4.76 | [S16] |
| POE/GNs | FDM 3D printing | 35 | 23 | [S17] |
| POM/GNs | Compression molding | 44.7 | 48 | [S18] |
| Epoxy/BaM/GNs | Solvent-free resin blending | 17.2 | 10 | [S19] |
| **PLA@GNs** | **FDM 3D printing** | **41.2** | **20** | **This work** |

(GNs: graphene nanosheets, PU: polyurethane, PMMA: methyl methacrylate, Ni: nickel, CNF: cellulose nanofiber, PI: polyimide, PS: polystyrene, HDPE: high-density polyethylene, PDMS: polydimethylsiloxane, POE: polyolefin elastomer, POM: polyoxymethylene, BaM: barium hexaferrite.)


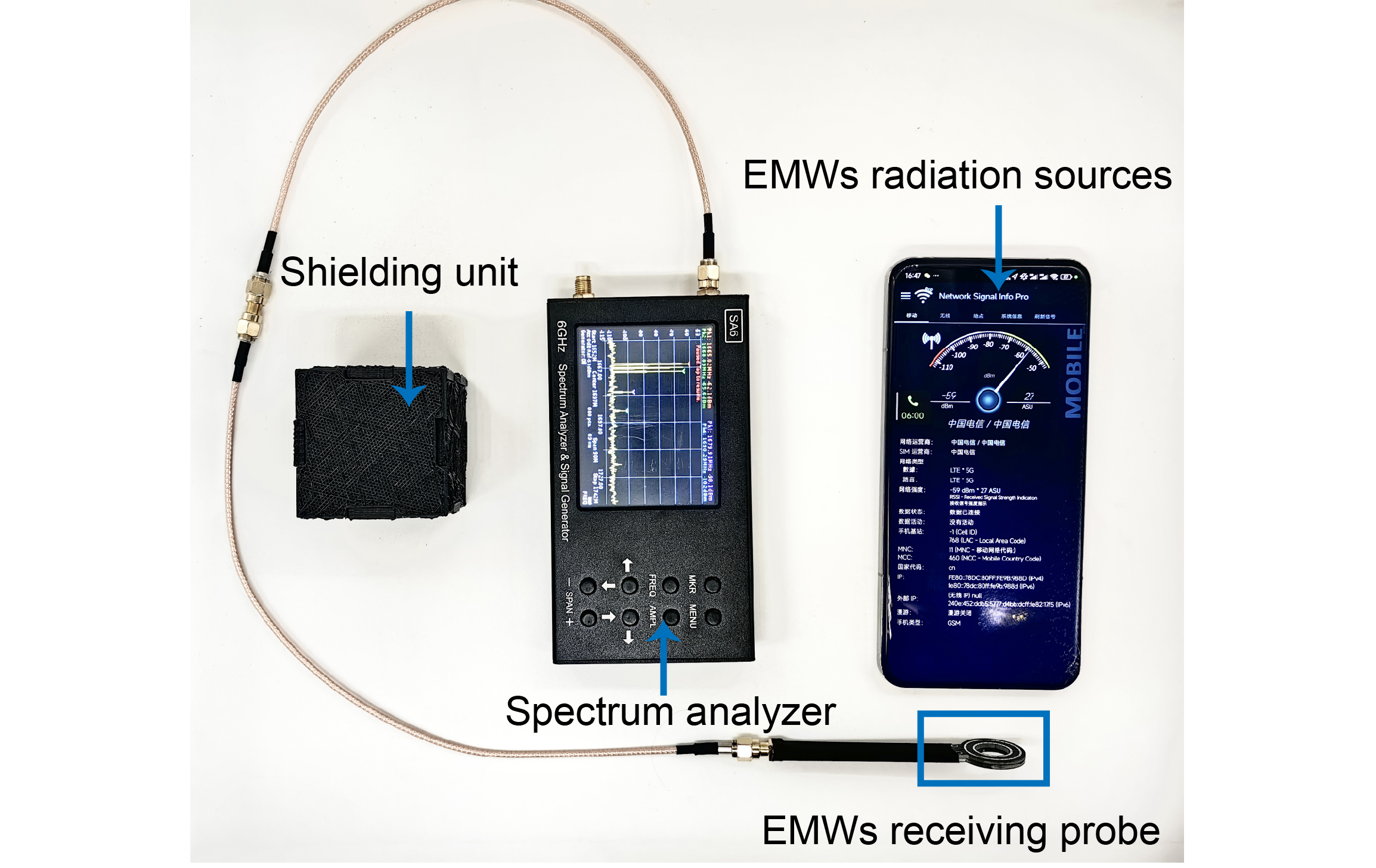


**Fig. S16** The as-manufactured EMWs signal detection system


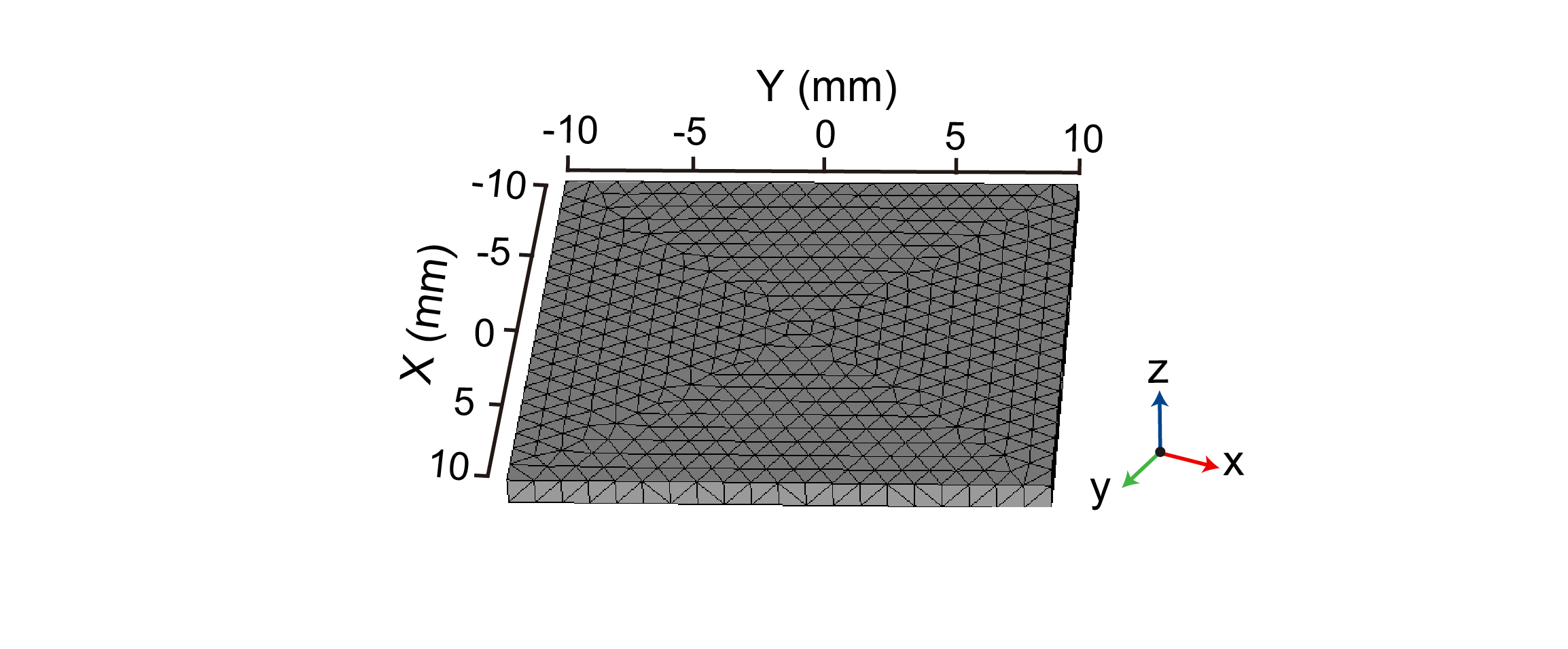


**Fig. S17** Mesh of 3D-printed module for electromagnetic simulation

**Supplementary References**

1. Y.-N. Gao, Y. Wang, T.-N. Yue, B. Zhao, R. Che et al., Superstructure silver micro-tube composites for ultrahigh electromagnetic wave shielding. Chem. Eng. J. **430**, 132949 (2022). <https://doi.org/10.1016/j.cej.2021.132949>
2. L. Zou, C. Lan, S. Zhang, X. Zheng, Z. Xu et al., Near-instantaneously self-healing coating toward stable and durable electromagnetic interference shielding. Nanomicro Lett. **13**(1), 190 (2021). <https://doi.org/10.1007/s40820-021-00709-0>
3. Q. Lv, X. Tao, S. Shi, Y. Li, N. Chen, From materials to components: 3D-printed architected honeycombs toward high-performance and tunable electromagnetic interference shielding. Compos. Part B Eng. **230**, 109500 (2022). <https://doi.org/10.1016/j.compositesb.2021.109500>
4. B.P. Heller, D.E. Smith, D.A. Jack, Planar deposition flow modeling of fiber filled composites in large area additive manufacturing. Addit. Manuf. **25**, 227–238 (2019). <https://doi.org/10.1016/j.addma.2018.10.031>
5. S. Bakrani Balani, F. Chabert, V. Nassiet, A. Cantarel, Influence of printing parameters on the stability of deposited beads in fused filament fabrication of poly(lactic) acid. Addit. Manuf. **25**, 112–121 (2019). <https://doi.org/10.1016/j.addma.2018.10.012>
6. S. Sharma, M. Goswami, A. Deb, B. Padhan, S. Chattopadhyay, Structural deformation/instability of the co-extrudate rubber profiles due to die swell: Experimental and CFD studies with 3D models. Chem. Eng. J. **424**, 130504 (2021). <https://doi.org/10.1016/j.cej.2021.130504>
7. S.G. Hatzikiriakos, Wall slip of molten polymers. Prog. Polym. Sci. **37**(4), 624–643 (2012). <https://doi.org/10.1016/j.progpolymsci.2011.09.004>
8. A. Lewandowski, K. Wilczyński, Global modeling of single screw extrusion with slip effects. Int. Polym. Process. **34**(1), 81–90 (2019). <https://doi.org/10.3139/217.3653>
9. M. Zhang, C. Huang, S. Sun, Y. Jia, The finite element simulation of polymer coextrusion based on the slip boundary. Polym. Plast. Technol. Eng. **48**(7), 754–759 (2009). <https://doi.org/10.1080/03602550902824648>
10. Y.-K. Li, W.-J. Li, Z.-X. Wang, P.-Y. Du, L. Xu et al., High-efficiency electromagnetic interference shielding and thermal management of high-graphene nanoplate-loaded composites enabled by polymer-infiltrated technique. Carbon **211**, 118096 (2023). <https://doi.org/10.1016/j.carbon.2023.118096>
11. Y. Pan, B. Yang, N. Jia, Y. Yang, Y. Wang et al., Polymethyl methacrylate (PMMA) nanocomposites containing graphene nanoplatelets decorated with nickel nanoparticles for electromagnetic interference (EMI) shielding and thermal management applications. Macromol. Mater. Eng. **307**(9), 2200220 (2022). <https://doi.org/10.1002/mame.202200220>
12. G. Wang, X. Liao, J. Yang, W. Tang, Y. Zhang et al., Frequency-selective and tunable electromagnetic shielding effectiveness *via* the sandwich structure of silicone rubber/graphene composite. Compos. Sci. Technol. **184**, 107847 (2019). <https://doi.org/10.1016/j.compscitech.2019.107847>
13. Y. Li, X. Pei, B. Shen, W. Zhai, L. Zhang et al., Polyimide/graphene composite foam sheets with ultrahigh thermostability for electromagnetic interference shielding. RSC Adv. **5**(31), 24342–24351 (2015). <https://doi.org/10.1039/c4ra16421k>
14. Y. Guo, L. Pan, X. Yang, K. Ruan, Y. Han et al., Simultaneous improvement of thermal conductivities and electromagnetic interference shielding performances in polystyrene composites *via* constructing interconnection oriented networks based on electrospinning technology. Compos. Part A Appl. Sci. Manuf. **124**, 105484 (2019). <https://doi.org/10.1016/j.compositesa.2019.105484>
15. M. Hamidinejad, B. Zhao, A. Zandieh, N. Moghimian, T. Filleter et al., Enhanced electrical and electromagnetic interference shielding properties of polymer–graphene nanoplatelet composites fabricated *via* supercritical-fluid treatment and physical foaming. ACS Appl. Mater. Interfaces **10**(36), 30752–30761 (2018). <https://doi.org/10.1021/acsami.8b10745>
16. C. Jiang, C. Hao, C. Zi, J. Li, W. Liu et al., Electrical and thermal conductive composites with thermal management and electromagnetic shielding enhanced by 3D network. Compos. Sci. Technol. **265**, 111135 (2025). <https://doi.org/10.1016/j.compscitech.2025.111135>
17. Q. Lv, Z. Peng, Y. Meng, H. Pei, Y. Chen et al., Three-dimensional printing to fabricate graphene-modified polyolefin elastomer flexible composites with tailorable porous structures for electromagnetic interference shielding and thermal management application. Ind. Eng. Chem. Res. **61**(45), 16733–16746 (2022). <https://doi.org/10.1021/acs.iecr.2c03086>
18. J. Li, Y. Wang, T.-N. Yue, Y.-N. Gao, Y.-D. Shi et al., Robust electromagnetic interference shielding, joule heating, thermal conductivity, and anti-dripping performances of polyoxymethylene with uniform distribution and high content of carbon-based nanofillers. Compos. Sci. Technol. **206**, 108681 (2021). <https://doi.org/10.1016/j.compscitech.2021.108681>
19. R.K. Bheema, A.K. Ojha, A.V. Praveen Kumar, K.C. Etika, Synergistic influence of barium hexaferrite nanoparticles for enhancing the EMI shielding performance of GNP/epoxy nanocomposites. J. Mater. Sci. **57**(19), 8714–8726 (2022). <https://doi.org/10.1007/s10853-022-07214-8>
